# Global, Population and Genetic Evidence on the Relationships Between Immune‐Mediated Inflammatory Disease and Cancer Risk

**DOI:** 10.1002/cai2.70048

**Published:** 2026-01-19

**Authors:** Xuesi Dong, Jiaxin Xie, Zilin Luo, Hao Hong, Chenran Wang, Yadi Zheng, Xiaoyue Shi, Zeming Guo, Xiaolu Chen, Yongjie Xu, Wei Cao, Fei Wang, Dong Hang, Sipeng Shen, Fengwei Tan, Ni Li

**Affiliations:** ^1^ Office of Cancer Screening, National Cancer Center/National Clinical Research Center for Cancer/Cancer Hospital Chinese Academy of Medical Sciences and Peking Union Medical College Beijing China; ^2^ Chinese Academy of Medical Sciences Key Laboratory for National Cancer Big Data Analysis and Implement Chinese Academy of Medical Sciences and Peking Union Medical College Beijing China; ^3^ Department of Biostatistics, School of Public Health Nanjing Medical University Nanjing Jiangsu China; ^4^ Jiangsu Key Lab of Cancer Biomarkers, Prevention and Treatment, Jiangsu Collaborative Innovation Center for Cancer Personalized Medicine Nanjing Medical University Nanjing Jiangsu China; ^5^ Changzhou Institute for Advanced Study of Public Health Nanjing Medical University Changzhou Jiangsu China; ^6^ Department of Epidemiology and Biostatistics, Jiangsu Key Lab of Cancer Biomarkers, Prevention and Treatment, Collaborative Innovation Center for Cancer Personalized Medicine, School of Public Health Nanjing Medical University Nanjing Jiangsu China; ^7^ Department of Thoracic Surgery, National Cancer Center/National Clinical Research Center for Cancer/Cancer Hospital Chinese Academy of Medical Sciences and Peking Union Medical College Beijing China

**Keywords:** cancer, epidemiology, evidence triangulation, immune‐mediated inflammatory diseases

## Abstract

**Background:**

Immune‐mediated inflammatory disease (IMID) and cancer share underlying mechanisms. We aimed to comprehensively evaluate the associations between IMIDs and cancers from global, population and genetic perspectives.

**Methods:**

A triangulation framework was employed to assess the association between IMIDs and cancers, using the Global Burden of Disease Study (2012–2021) to analyse six IMIDs and 33 cancers. The UK Biobank (UKBB) prospective cohort was subsequently used to validate these associations, with hazard ratios (HRs) and 95% confidence intervals (CIs) estimated by Cox proportional hazards models. Causal inference based on genetic instruments was performed in the FinnGen and UKBB to assess the potential causal effects between IMIDs and cancers.

**Results:**

IMIDs were positively associated with the occurrence of cancers from a global perspective. Moreover, 170 specific IMID‐cancer pairs revealed statistically significant associations. A total of 20 pairs of specific IMID‐cancer associations were further confirmed in the UKBB cohort. Among these, the five most pronounced associations included atopic dermatitis with Hodgkin lymphoma (HR = 12.56, 95% CI: 1.76–89.59), with ovarian cancer (HR = 5.65, 95% CI: 1.41–22.65) and with non‐Hodgkin lymphoma (HR = 5.11, 95% CI: 1.91–13.63); rheumatoid arthritis with Hodgkin lymphoma (HR = 3.85, 95% CI: 1.11–13.32); and psoriasis with Hodgkin lymphoma (HR = 3.43, 95% CI: 1.69–6.96). Additionally, a positive causal association between rheumatoid arthritis and Hodgkin lymphoma (inverse variance weighted OR = 1.31, 95% CI: 1.10–1.57) was observed.

**Conclusions:**

This study provides comprehensive evidence of the relationships between IMIDs and cancers from global, population and genetic perspectives and identifies 20 pairs of specific IMID‐cancer associations, thereby contributing to advancements in cancer prevention and control.

## Graphical abstract

Using a triangulation framework that synthesizes findings across global, population and genetic perspectives, this work comprehensively establishes associations between six immune‐mediated inflammatory diseases (IMIDs) and 33 cancers, identifying 170 statistically significant IMID‐cancer pairs, validating 20 in the UKBB cohort and uncovering a causal link between rheumatoid arthritis and Hodgkin lymphoma.

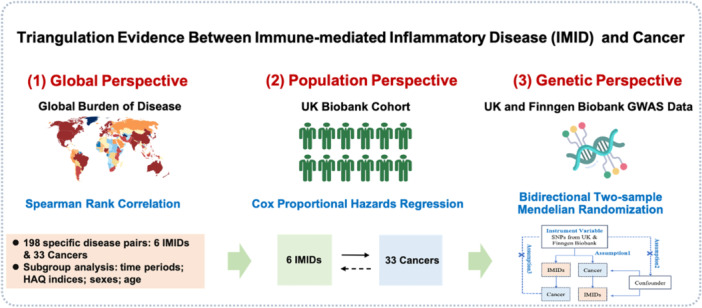

**Abbreviations**
BMI: body mass index
CI: confidence interval
FDR: false discovery rate
GBD: Global Burden of Disease
GWAS: Genome‐Wide Association Study
HAQ: Healthcare Access and Quality
HR: hazard ratio
ICD‐10: International Classification of Diseases, Tenth Revision
IMID: Immune‐Mediated Inflammatory Disease
IVW: inverse variance weighted
MR: Mendelian Randomization
OR: odds ratio
UKBB: UK Biobank

## 1 Introduction

Immune‐mediated inflammatory disease (IMID) is a cluster of chronic conditions involving inappropriate or excessive immune responses triggered or accompanied by acute or chronic inflammation [1]. IMID includes asthma, atopic dermatitis, inflammatory bowel disease, multiple sclerosis, psoriasis and rheumatoid arthritis [2]. In 2019, the global incidence of IMID reached approximately 68 million people, with an age‐standardized rate of 909 per 100,000 people [2]. Cancer is one of the leading causes of death worldwide [3]. According to the latest data from GLOBOCAN, there were 19.3 million new cancer cases and 10.0 million cancer‐related deaths in 2020 [4].

IMID and cancer share overlapping risk factors, including genetic, environmental and behavioural factors [2]. Previous cohort studies have demonstrated a greater cancer risk in patients with various IMIDs than in the general population [5, 6]. Studies at the molecular level have revealed the intricate interaction mechanisms among immunity, inflammation and cancer [7, 8]. Over the past decade, the generalization of immunotherapy and anti‐inflammatory agents has led to promising improvements in cancer prevention and treatment [9]. For example, the use of monoclonal antibodies targeting CTLA‐4 could improve the 3‐year survival rate of patients with melanoma by approximately 12% [10, 11]. Individuals who regularly used nonsteroidal anti‐inflammatory drugs, for example, aspirin, exhibited a 27% lower risk of colorectal cancer than nonusers [12], stressing the importance of modulating human immune and inflammation status in cancer treatment. However, most prior studies focus on single IMIDs or single cancer types, and comprehensive and integrative evidence across multiple dimensions (global, population and genetic) remains lacking.

Evidence triangulation, a methodological approach that synthesizes findings across diverse approaches, offers a framework for enhancing the credibility and validity of research findings [13]. On the basis of this theory, we aim to conduct a comprehensive analysis of the associations and causalities between IMID and cancer incidence from global, population and genetic perspectives and provide new insight into cancer prevention and control.

## 2 Methods

### 2.1 Study Design

**Figure 1:**
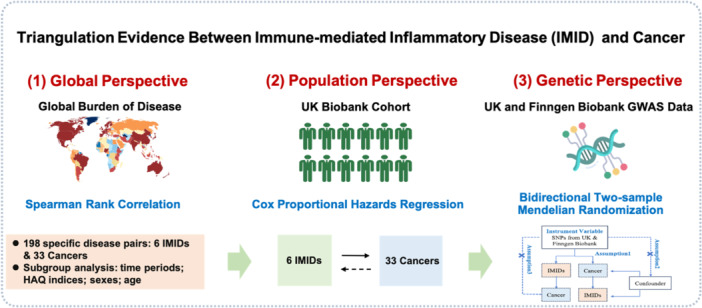
Flowchart of the research process.

An evidence triangulation framework, which synthesizes findings across three strategies, including global, population and genetic perspectives, was employed to systematically evaluate the associations between IMIDs and cancers [13]. The flowchart describing this method is shown in Figure 1. First, a comprehensive analysis of global patterns was conducted using data from the Global Burden of Disease (GBD) study database. Subgroup analyses were performed across different time periods, Healthcare Access and Quality (HAQ) indices, sexes and age groups. Second, we investigated the observed associations in a prospective population‐based cohort, the UK Biobank (UKBB). Additionally, bidirectional two‐sample Mendelian randomization (MR) analyses were performed using summary‐level statistics from GWAS of IMIDs and cancers in the FinnGen biobank and UKBB.

### 2.2 Study Database and Population‐Based Cohorts

#### 2.2.1 Global‐Level Data From the GBD Study

The GBD Study (2021) comprehensively explored 371 diseases and injuries, conducting an in‐depth analysis of historical data from 204 countries and territories worldwide, spanning from 1990 to 2021. The research methodology has been extensively described elsewhere [14]. Users of the research data can access it through the online platform of the Global Health Data Exchange [15]. We extracted the age‐standardized incidence rates of IMIDs and cancers from 2012 to 2021 for 204 countries and territories globally. Additionally, age‐standardized incidence rates were obtained by sex and crude values across different age groups (0–4, 5–14, 15–49, 50–74, 75–84 and 85 years and above) in 2021. The HAQ index, which is calculated on the basis of ranges of risk‐standardized death rates and mortality‐to‐incidence ratios in the GBD dataset, was used to measure the accessibility and quality of health care services across different countries [16].

#### 2.2.2 Population‐Level Data From UKBB

The UKBB is a prospective population‐based cohort study that enroled over 500,000 participants aged 40–69 years and was initiated in the United Kingdom [17]. Participants who withdrew informed consent were excluded from the analysis. All participants provided written informed consent. The following covariates were included in the analysis: age (years), sex (male, female), body mass index (BMI, kg/m^2^), smoking history (nonsmokers, current smokers, ex‐smokers), alcohol intake (never, special occasions only, one to three times a month, once or twice a week, three or four times a week, almost daily or daily) and family history of cancer (yes, no). Details of the cohort and measurements are available in the UK Biobank Data Showcase (http://biobank.ctsu.ox.ac.uk/crystal/). The UKBB study was approved by the Research Ethics Committee (reference 11/NW/0382).

#### 2.2.3 Genetic‐Level Data From the FinnGen Biobank and UKBB

Summary‐level GWAS data were obtained from the most recent large‐scale studies, including the FinnGen biobank (*N* = 500,348) and UKBB (*N* = 487,409). IMIDs GWAS data, which include information about asthma, atopic dermatitis, inflammatory bowel disease, multiple sclerosis, psoriasis and rheumatoid arthritis, were derived from the FinnGen R11 release. Summary‐level GWAS statistics were estimated by UKBB using a testing framework based on generalized mixed model associations. Quality control of the input genetic data used in the GWAS has been described elsewhere [18, 19]. We retained variants that had a MAF > 0.1% and INFO scores > 0.3. For each GWAS conducted for each phenotype, we adjusted for age, sex, age^2^, age × sex and the first 10 principal components. Covariates, including age and age^2^, the first 10 principal components, were adjusted for sex‐specific data. All summary data were publicly available, and no further ethical approval was necessary. All analyses were conducted with REGENIE v3.5 software.

### 2.3 Definitions of Exposure and Outcomes

The IMID considered in our study encompasses asthma, inflammatory bowel disease, multiple sclerosis, rheumatoid arthritis, psoriasis and atopic dermatitis [2]. The six IMIDs included in this study were selected based on the disease burden (accounting for 99.4% newly diagnosed IMID cases in 2019), clinical relevance and prior evidence suggesting the emergence of novel drugs and treatment strategies over the past few decades [2]. Both overall cancer and 33 specific cancer groups were considered, as detailed in Supporting Information S1: Data S1 [14, 20]. The diagnosis code for the aforementioned disease followed the International Classification of Diseases, Tenth Revision (ICD‐10) [14, 20].

For the UKBB cohort, baseline data (2006–2010) were the starting point. Individuals with prevalent IMIDs reported at baseline were classified into the exposure group. Participants were followed from the recruitment date until the earliest occurrence of cancer diagnosis, death, loss to follow‐up or the end of the study period (31 October 2022). Reverse associations were also investigated by considering baseline cancer cases as the exposure group and evaluating subsequent IMID incidence. The diagnosis date was defined as the first record of a corresponding ICD‐10 code in the participants' inpatient records.

### 2.4 Statistical Analysis

To investigate the correlation between IMIDs and cancers in GBD, Spearman's rank correlation analysis was conducted on the age‐standardized incidence rates of both diseases at the global level. Hazard ratios (HRs) and 95% confidence intervals (CIs) were subsequently estimated using Cox proportional hazards regression models. These analyses were conducted for significant disease pairs identified from the GBD database. The following three models were constructed for the UKBB cohort: Model 1, unadjusted; Model 2, adjusted for age and sex; Model 3, adjusted for age, sex, BMI, smoking history, alcohol intake and family history of cancer. IMID‐cancer pairs were confirmed if they showed statistically significant associations in both the GBD and UKBB datasets (*p* < 0.05) and demonstrated a consistent direction of association across the two datasets. To account for multiple testing, *p*‐values from UKBB analyses were further adjusted using the Benjamini–Hochberg false discovery rate (FDR) method. Missing covariate data were handled using multiple imputation. To reduce potential surveillance bias, sensitivity analyses were performed by excluding IMID diagnoses that occurred within 1 year after cancer diagnosis.

Bidirectional two‐sample MR analysis was performed to investigate the causal relationship between IMID and cancer risk. We used the 1000 genomes reference panel to obtain linkage disequilibrium. The strength of the genetic instruments was assessed by calculating the *F*‐statistic. Only instruments with an *F*‐statistic > 10 were selected, indicating sufficient instrument strength and validity. The inverse variance weighted (IVW) [21] method was used as the main method to estimate the odds ratio (OR) and 95% confidence interval (CI); detailed information is described in Supporting Information S1: Data S2. Multiple sensitivity analyses, including MR‐Egger [22], weighted mode [23], weighted median [24] and maximum likelihood approaches [25], were used as complementary analyses to examine the robustness of the results. MR‐PRESSO was performed to account for potential violations of the instrumental variable assumptions [26, 27].

Statistical analysis and visualization were performed using R software (version 4.1.3). All statistical tests were two‐sided. This study strictly adheres to the Guidelines for Accurate and Transparent Health Estimates Reporting (GATHER) statement [14] and to the Strengthening the Reporting of Observational Studies in Epidemiology using Mendelian Randomization (STROBE‐MR) guidelines [28, 29].

## 3 Results

### 3.1 Global Correlation Between IMIDs and Cancer Risk

**Figure 2:**
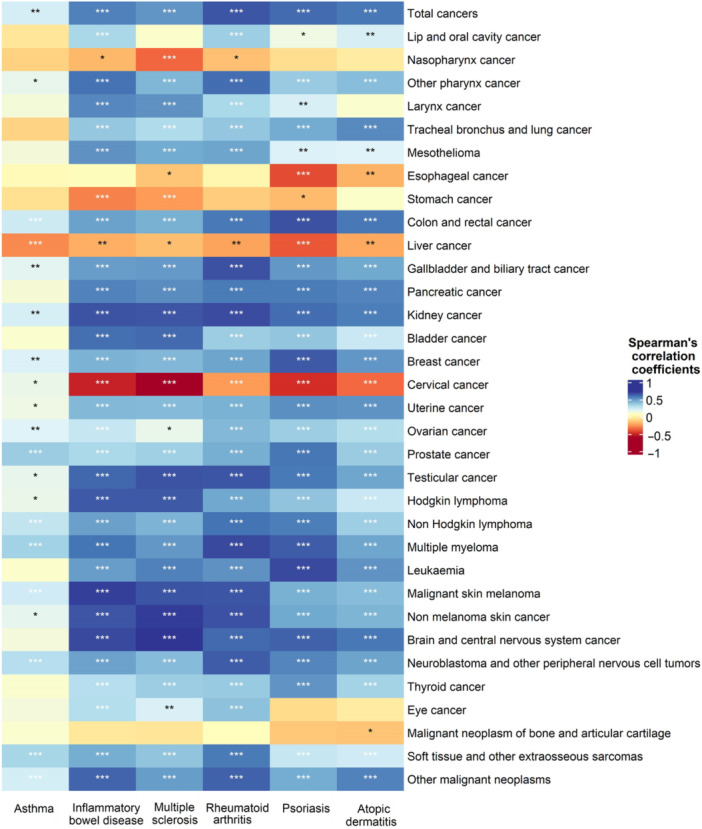
Spearman rank correlations between age‐standardized incidence rate of immune‐mediated inflammatory diseases and cancers in Global Burden of Disease 2021. Significance is indicated as follows: ***p < 0.001, **p < 0.01, *p < 0.05.

All six IMIDs had risks that were significantly positively correlated with cancer risk. Strong correlations were observed with rheumatoid arthritis (Rho = 0.67, *p* < 0.001) and psoriasis (Rho = 0.62, *p* < 0.001). Moderate correlations were noted with atopic dermatitis (Rho = 0.59, *p* < 0.001), inflammatory bowel disease (Rho = 0.57, *p* < 0.001) and multiple sclerosis (Rho = 0.52, *p* < 0.001), whereas a weak correlation was found with asthma (Rho = 0.23, *p* = 0.001). Among the 198 specific IMID‐cancer pairs (six from the IMID group and 33 from the cancer group), 149 (75.3%) were significantly positively correlated (*p* < 0.05). The strongest associations were found between multiple sclerosis and brain and central nervous system cancer (Rho = 0.73, *p* < 0.001), non‐melanoma skin cancer (Rho = 0.72, *p* < 0.001) and testicular cancer (Rho = 0.67, *p* < 0.001). Additionally, rheumatoid arthritis was strongly associated with multiple myeloma (Rho = 0.69, *p* < 0.001), kidney cancer (Rho = 0.69, *p* < 0.001), gallbladder and biliary tract cancer (Rho = 0.68, *p* < 0.001) and non‐melanoma skin cancer (Rho = 0.67, *p* < 0.001). Inflammatory bowel disease was strongly correlated with malignant skin melanoma (Rho=0.67, *p* < 0.001), brain and central nervous system cancer (Rho = 0.69, *p* < 0.001) and kidney cancer (Rho = 0.67, *p* < 0.001). Psoriasis was strongly correlated with leukaemia (Rho = 0.69, *p* < 0.001) and colon and rectal cancer (Rho = 0.68, *p* < 0.001). Conversely, 21 (10.6%) IMID‐cancer pairs presented significant negative correlations. For example, multiple sclerosis (Rho = −0.60, *p* < 0.001), inflammatory bowel disease (Rho = −0.51, *p* < 0.001) and psoriasis were moderately negatively correlated with cervical cancer (Rho = −0.47, *p* < 0.001; Figure 2).

### 3.2 Subgroup Analysis of the Correlations Between IMIDs and Cancers

From 2012 to 2021, the correlations between the six IMIDs and cancer incidence rates remained stable. In 2012, rheumatoid arthritis (Rho = 0.68, *p* < 0.001), psoriasis (Rho = 0.64, *p* < 0.001), atopic dermatitis (Rho = 0.63, *p* < 0.001), inflammatory bowel disease (Rho = 0.62, *p* < 0.001), multiple sclerosis (Rho = 0.56, *p* < 0.001) and asthma (Rho = 0.17, *p* = 0.014) were positively correlated with cancer (Supporting Information S1: Figure S1).

In countries with high HAQ indices, inflammatory bowel disease (Rho = 0.72, *p* < 0.001) and multiple sclerosis (Rho = 0.50, *p* < 0.001) were positively correlated with cancer. The correlation between asthma and cancer was significant only in low‐HAQ countries (Rho = 0.21, *p* = 0.037). In both high‐ and low‐HAQ index countries, atopic dermatitis, psoriasis and RA were positively correlated with cancer (Supporting Information S1: Figure S2).

In both males and females, all six IMIDs were positively correlated with cancer, with varying degrees of association. Among males, psoriasis was strongly associated with cancer (Rho = 0.60, *p* < 0.001); inflammatory bowel disease (Rho = 0.57, *p* < 0.001), rheumatoid arthritis (Rho = 0.55, *p* < 0.001), atopic dermatitis (Rho = 0.54, *p* < 0.001) and multiple sclerosis (Rho = 0.52, *p* < 0.001) were moderately associated with cancer, while the associations between asthma and cancer were weaker (Rho = 0.21, *p* = 0.003). Conversely, in women, rheumatoid arthritis (Rho = 0.65, *p* < 0.001) and psoriasis (Rho = 0.61, *p* < 0.001) were strongly associated with cancer. Moderate associations were observed for atopic dermatitis (Rho = 0.59, *p* < 0.001), inflammatory bowel disease (Rho = 0.53, *p* < 0.001) and multiple sclerosis (Rho = 0.49, *p* < 0.001), whereas asthma was weakly associated with cancer (Rho = 0.25, *p* < 0.001) (Supporting Information S1: Figures S3 and S4).

A positive correlation was observed between asthma and cancer in the 0–4 years age group (Rho = 0.17, *p* = 0.013) and 15–49 years age group (Rho = 0.18, *p* = 0.008), with no significant association in the 5–14 years range (Rho = 0.12, *p* = 0.084) and an inverse correlation after 50 years (Rho = −0.33, *p* < 0.001). The associations between multiple sclerosis, psoriasis and rheumatoid arthritis with cancer tended to decrease with age; however, multiple sclerosis and rheumatoid arthritis were rare in those under 4 years of age, and no significant associations were detected between psoriasis and cancer during this period (Rho = 0.13, *p* = 0.059). The association between multiple sclerosis and cancer was no longer significant after 75 years (Rho = 0.043, *p* = 0.540). Atopic dermatitis and inflammatory bowel disease were consistently positively correlated with cancer across various age brackets (Supporting Information S1: Figure S5).

### 3.3 Population‐Level Associations Between IMIDs and Cancer Risk

**Figure 3:**
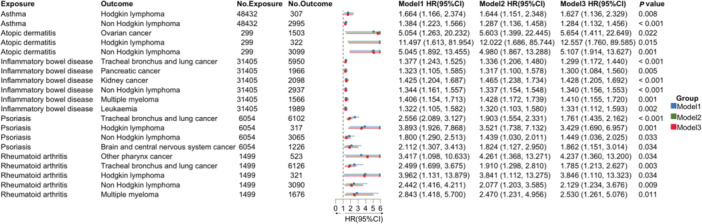
Hazard Ratios between specific immune‐mediated inflammatory diseases and cancers in the UK Biobank. Only significant immune‐mediated inflammatory disease and cancer pairs in the Global Burden of Disease and UK Biobank are shown. Model 1: Unadjusted Cox proportional hazards model; Model 2: Cox proportional hazards model adjusted for age and sex; Model 3: Cox proportional hazards model adjusted for age, sex, BMI, smoking history, alcohol intake, and family history of cancer. *p* value indicates the maximum *p*‐value result among Model 1, Model 2 and Model 3. CI, confidence interval; HR, hazard ratio.

Overall, patients with IMIDs had a greater risk of developing cancer (HR = 1.07, 95% CI: 1.05–1.10) (Supporting Information S1: Table S1). Stratified analysis by IMID type revealed that psoriasis (HR = 1.13, 95% CI: 1.05–1.22), asthma (HR = 1.04, 95% CI: 1.01–1.07) and inflammatory bowel disease (HR = 1.04, 95% CI: 1.01–1.07) were significantly associated with an increased risk of cancer. For the association between IMIDs and the risk of specific cancers identified in the GBD database (170 specific IMID‐cancer pairs), 20 out of 170 were confirmed in the UKBB cohort (Figure 3). Among these patients, those with IMID were most commonly associated with an increased risk of haematological malignancies (12/20), followed by those with cancers of the respiratory system (4/20) and other malignancies. The five strongest associations were atopic dermatitis and Hodgkin lymphoma (HR = 12.56, 95% CI: 1.76–89.59), ovarian cancer (HR = 5.65, 95% CI: 1.41–22.65) and non‐Hodgkin lymphoma (HR = 5.11, 95% CI: 1.91–13.63); rheumatoid arthritis and Hodgkin lymphoma (HR = 3.85, 95% CI: 1.11–13.32); and psoriasis and Hodgkin lymphoma (HR = 3.43, 95% CI: 1.69–6.96). All significant IMID‐cancer pairs presented a greater risk of cancer (HR > 1).

In the reverse association analysis, cancer was associated with an increased risk of IMID (HR = 1.03, 95% CI: 1.00–1.06) (Supporting Information S1: Table S2). Overall, 29 cancer‐IMID pairs demonstrated significant associations (Supporting Information S1: Figure S6). Among the observed associations, the five pairs with the highest HRs were mesothelioma and atopic dermatitis (HR = 10.45, 95% CI: 1.45–75.40), followed by multiple myeloma and atopic dermatitis (HR = 9.21, 95% CI: 2.93–28.93), ovarian cancer and atopic dermatitis (HR = 4.83, 95% CI: 1.19–19.58), colon and rectal cancer and atopic dermatitis (HR = 3.62, 95% CI: 1.79–7.34) and multiple myeloma and inflammatory bowel disease (HR = 3.13, 95% CI: 2.64–3.71). Notably, liver cancer was the only cancer found to be associated with a decreased risk of asthma (HR = 0.37, 95% CI: 0.23–0.59). Sensitivity analyses excluding IMID diagnoses that occurred within 1 year after cancer diagnosis showed consistent results with the main analysis (Supporting Information S1: Table S3).

Several IMIDs and cancers exhibit bidirectional associations. For example, rheumatoid arthritis and other pharynx cancers have reciprocal relationships, whereas inflammatory bowel disease is bidirectionally associated with multiple malignancies, including tracheal, bronchial and lung cancer; pancreatic cancer; non‐Hodgkin lymphoma; multiple myeloma and leukaemia. Additional bidirectional associations were found between atopic dermatitis and ovarian cancer, as well as psoriasis and non‐Hodgkin lymphoma.

### 3.4 Causal Links of Genetic Liability to IMIDs and Cancers

**Figure 4:**
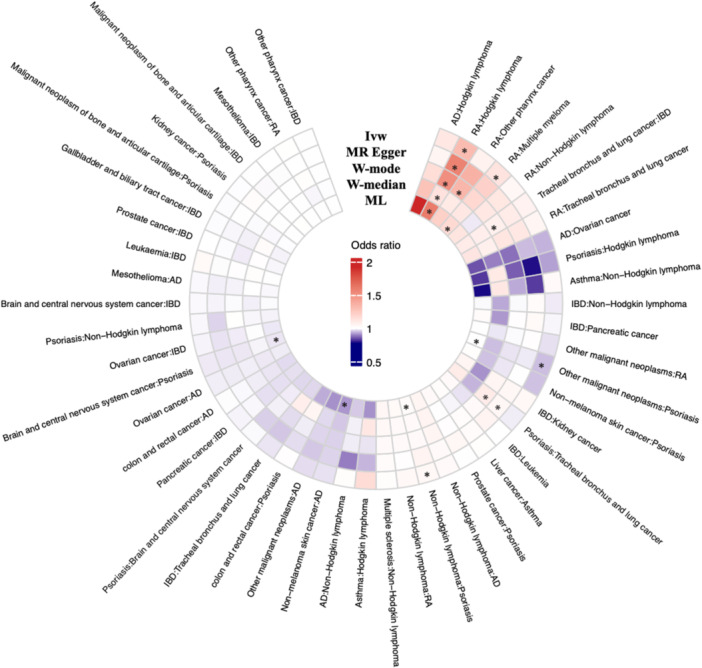
Summary of causal effects between immune‐mediated inflammatory diseases and cancers in Mendelian randomization. The disease pairs are presented as exposure: Outcome. Asterisk (*) indicates significant results (*p*‐value < 0.05). AD, atopic dermatitis; IBD, inflammatory bowel disease; IVW, inverse variance weighted; ML, maximum likelihood; RA, rheumatoid arthritis; W‐median, weighted median; W‐mode, weighted mode.

Two‐sample MR analysis was performed on 49 disease pairs that showed significant associations at both the global and population levels (Figure 4, Supporting Information S1: Table S4). Only one positive association, rheumatoid arthritis‐Hodgkin lymphoma (OR_IVW_ = 1.31, 95% CI: 1.10–1.57), was noted with IMID exposure (Supporting Information S1: Table S5). No reverse causal association was identified. Sensitivity analyses, including MR–Egger, weighted mode, weighted median and maximum likelihood methods, consistently reinforced the robustness of these findings under various assumptions. No significant heterogeneity (*p* = .512) or horizontal pleiotropy (*p* = .245) was observed in the association between rheumatoid arthritis and Hodgkin lymphoma. Scatter and funnel plots revealed no evidence of outliers or weak instrument biases (Supporting Information S1: Figure S7). No positive association was identified with cancer exposure.

## 4 Discussion

In this study, we provide the first comprehensive relationships between IMID and cancer by integrating evidence from global, population and genetic perspectives. In total, 20 pairs of IMID‐cancer associations were observed in this study. Moreover, the association between rheumatoid arthritis and Hodgkin lymphoma was validated to be causal.

This study systematically compared the global correlation strengths between IMIDs and various cancers, with the strongest positive correlations observed for brain and central nervous system cancer, non‐melanoma skin cancer and kidney cancer. Previous studies have observed that individuals with AD may have a 2.52‐fold risk of developing brain cancer compared with the general population [30]. A meta‐analysis indicated that the risk of non‐melanoma skin cancer in psoriasis patients increased by 71%, and in severe psoriasis patients, the risk increased by 144% [31]. Another meta‐analysis of cohort studies revealed an 86% increase in the risk of kidney cancer in patients with atopic dermatitis [32]. Population‐based study revealed increased risks of basal cell carcinoma (22%) and squamous cell carcinoma (88%) in patients with rheumatoid arthritis not receiving biologic agents, with additional risks observed in those treated with TNF inhibitors [33]. These patterns may reflect shared mechanisms such as chronic systemic inflammation, immune dysregulation and immunosuppressive therapies. From a translational perspective, these findings emphasize the importance of prioritizing cancer screening and monitoring strategies for high‐risk IMID patients, as well as long‐term surveillance of immune‐related complications in cancer survivors.

Our study quantified the disparity in the association between IMID and cancer under different HAQs on a global scale. In addition to atopic dermatitis, psoriasis, and rheumatoid arthritis, inflammatory bowel disease and multiple sclerosis require prioritized attention in high‐HAQ countries, whereas asthma emerges as a critical concern in countries with lower HAQ levels. This difference may stem from contrasting healthcare accessibility and quality; differences in disease treatment, medications, complication prevention and surveillance; and varying levels of cancer screening implementation. In the lower HAQ countries, additional factors such as environmental exposures (e.g., air pollution and occupational hazards) may further contribute to the observed association. This finding provides evidence for health resource allocation strategies tailored to healthcare capacity.

IMID patients were associated with a greater risk of developing cancer after adjustment for common cancer risk factors such as smoking history, alcohol consumption and family history of cancer. Haematological malignancies were among the cancers most extensively associated with different IMIDs, such as asthma, atopic dermatitis, inflammatory bowel disease, psoriasis and rheumatoid arthritis. A recent meta‐analysis reported a 2‐ to 12‐fold increased risk of lymphoma in rheumatoid arthritis patients, which is consistent with our findings [34, 35]. Nongenetic factors, such as secondary inflammation due to autoimmune stimulation, cytokine and chemokine release, human immunodeficiency virus (Epstein‐Barr virus) infections and treatment effects, might also play a substantial role [34, 36, 37, 38]. Genetically, our study provides further evidence supporting a causal relationship, showing that rheumatoid arthritis patients have a 31% greater risk of developing Hodgkin lymphoma. A possible explanation might be genetic variation in the major histocompatibility complex, especially in the human leukocyte antigen (HLA) Class II region [39, 40, 41]. A previous study demonstrated that the HLA‐DRB1*0101/HLA‐DQA1*0101 allele type might be a potential shared genetic susceptibility [39]. These findings offer critical insights into the underlying mechanisms linking rheumatoid arthritis and Hodgkin lymphoma and provide a basis for targeted prevention and management strategies in high‐risk populations.

Among the USPSTF‐recommended screening cancers, our study highlights that IMIDs, including inflammatory bowel disease, psoriasis and rheumatoid arthritis, are associated with a greater risk of tracheal bronchus and lung cancer. The observed risk estimates are consistent with those of prior studies, which reported a 1.64‐, 1.53‐ and 1.26‐fold increased risk of lung cancer in individuals with rheumatoid arthritis, inflammatory bowel disease and psoriasis, respectively [6, 35, 42]. By evidence triangulation, our study provides more comprehensive evidence of the relationship between IMIDs and lung cancer risk, suggesting that certain IMIDs might serve as potential clinical surrogate markers for high‐risk population identification in lung cancer screening [43].

Furthermore, our study also revealed reverse associations, whereby certain cancers were associated with an increased risk of specific IMIDs, including atopic dermatitis. Multiple mechanisms could partially explain these patterns, including chronic inflammation promoting autoimmune responses, shared genetic susceptibility, development of autoimmune paraneoplastic disease and immune‐related adverse events induced by treatment [44, 45, 46]. Furthermore, our study revealed that liver cancer was inversely associated with the risk of developing asthma, which is consistent with findings from a Korean nationwide cohort [47]. This could be explained in part by hepatocellular carcinoma patients exhibiting irreversible damage and fibrosis in the cirrhotic liver, which then impairs innate immunity through reducing the synthesis and function of immune‐related proteins, potentially attenuating the allergy‐driven immune responses within the liver associated with allergic diseases [47, 48, 49, 50, 51, 52]. This highlights potential insights to guide the development of innovative therapies.

The main strength of this study lies in the application of a triangulation approach to comprehensively and quantitatively analyse the relationship between IMID and cancer incidence across a global, population and genetic scale. Leveraging data from large‐scale global association analysis enhances the generalizability and credibility of the findings. Population‐level data from a large prospective cohort minimized ecological fallacies, whereas MR analysis reduced biases from unmeasured confounders, providing insights into genetic predispositions for causal relationships.

Several limitations should be considered. First, IMID and cancer incidence rates are potentially underestimated in underdeveloped regions due to underdiagnosis and incomplete records in the GBD. Nonetheless, the underestimation of IMIDs and cancer incidence is likely similar, thereby minimally impacting the analytical results. Additionally, certain IMIDs and cancer risk dyads in the UKBB cohort demonstrated wide confidence intervals due to the relatively modest number of observations present. Third, while bidirectional MR analysis minimizes bias, the selection of instrumental variables remains a challenge for complex diseases. Multiple sensitivity analysis methods under different assumptions were considered to ensure the robustness and reliability of our findings. Besides, as most of our data were derived from European populations, caution is warranted when generalizing these findings to other ethnic groups and regions.

## 5 Conclusion

In conclusion, our findings provide comprehensive evidence that IMID is associated with the risk of cancer incidence. These findings indicate a potential commonality in the mechanism underlying specific IMIDs and cancers, highlighting the urgent need for integrated prevention and control strategies for cancer.

## Author Contributions

**Xuesi Dong:** conceptualization (lead), writing – review and editing (equal), writing – original draft (equal), supervision (equal), resources (lead), project administration (lead), data curation (equal). **Jiaxin Xie:** methodology (lead), software (lead), formal analysis (equal), visualization (lead), writing – original draft (lead), data curation (equal). **Zilin Luo:** formal analysis (lead), methodology (equal), visualization (equal), writing – original draft (equal), data curation (equal). **Hao Hong:** formal analysis (equal), methodology (equal), visualization (supporting), data curation (equal). **Chenran Wang:** validation (lead), writing – review and editing (supporting). **Yadi Zheng:** validation (equal), writing – review and editing (equal). **Xiaoyue Shi:** validation (equal), writing – review and editing (supporting). **Zeming Guo:** validation (equal), writing – review and editing (equal). **Xiaolu Chen:** validation (equal), writing – review and editing (equal). **Yongjie Xu:** writing – review and editing (supporting). **Wei Cao:** writing – review and editing (supporting). **Fei Wang:** writing – review and editing (supporting). **Dong Hang:** writing – review and editing (supporting). **Sipeng Shen:** writing – review and editing (equal), supervision (lead). **Fengwei Tan:** writing – review and editing (equal), supervision (equal). **Ni Li:** conceptualization (equal), writing – review and editing (lead), funding acquisition (lead), supervision (lead).

## Ethics Statement

The UK Biobank study received ethical approval from the Northwest Multicentre Research Ethics Committee (approval number: 11/NW/0382).

## Consent

All participants signed informed consent forms.

## Conflicts of Interest

The authors declare no conflicts of interest.

## Supporting information

Supporting materials‐final.

## Data Availability

This study has been conducted using the UK Biobank Resource under Application Number UKBB‐92675. Summary statistics for FinnGen biobank were extracted from https://www.finngen.fi/en; GBD dataset was obtained from https://ghdx.healthdata.org/gbd-2021.
