# Supplementary material for: Global, Population and Genetic Evidence on the Relationships Between Immune‐Mediated Inflammatory Disease and Cancer Risk
Source: Cancer Innov. 2026 Jan 19;5(1):e70048. doi: 10.1002/cai2.70048 (PMC12815612; doi:10.1002/cai2.70048)
Supplement: Supplementary file 1 — Supporting materials‐final. [file CAI2-5-e70048-s001.docx]

**Supplementary 1 International Classification of Diseases, Tenth Revision (ICD-10) for 6 immune-mediated inflammatory diseases and 33 cancers**

The cancer types and the corresponding ICD-10 codes considered in our study are as follows: lip and oral cavity cancer (C00–C07, C08–C08.9), nasopharynx cancer (C11–C11.9), other pharynx cancer (C09–C10.9, C12–C13.9), larynx cancer (C32–C32.9), tracheal, bronchus, and lung cancer (C33, C34–C34.92), mesothelioma (C45–C45.2, C45.7, C45.9), esophageal cancer (C15–C15.9), stomach cancer (C16–C16.9), colon and rectal cancer (C18–C19.0, C20, C21–C21.8), liver cancer (C22–C22.4, C22.7–C22.8), gallbladder and biliary tract cancer (C23, C24–C24.9), uterine cancer (C54–C54.3, C54.8–C54.9), ovarian cancer (C56–C56.2, C56.9), prostate cancer (C61–C61.9), testicular cancer (C62–C62.92), Hodgkin lymphoma (C81–C81.49, C81.7–C81.79, C81.9–C81.99), non-Hodgkin lymphoma (C82–C85.29, C85.7–C86.6, C96–C96.9), multiple myeloma (C88–C90.32), leukaemia (C91–C93.7, C93.9–C95.2, C95.7–C95.92), malignant skin melanoma (C43–C43.9), non-melanoma skin cancer (C44.01–C44.99), brain and central nervous system cancer (C70–C70.1, C70.9–C72.9), neuroblastoma and other peripheral nervous cell tumors (C47–C47.9), thyroid cancer (C73), eye cancer (C69–C69.92), malignant neoplasm of bone and articular cartilage (C40–C40.92, C41.0–C41.4, C41.8–C41.9), soft tissue and other extraosseous sarcomas (C49–C49.9), pancreatic cancer (C25–C25.9), kidney cancer (C64–C64.2, C64.9–C65.9), bladder cancer (C67–C67.9), breast cancer (C50–C50.629, C50.8–C50.929), cervical cancer (C53–C53.9), and other malignant neoplasms (C17–C17.9, C30–C30.1, C31–C31.9, C37–C37.0, C38–C38.8, C41, C44–C44.00, C48–C48.9, C4A, C51–C52, C57–C57.8, C58–C58.0, C60–C60.9, C63–C63.8, C66–C66.9, C68.0–C68.8, C74–C75.5, C75.8); The immune-mediated inflammatory diseases (IMIDs) considered and the corresponding ICD-10 codes are as follows: asthma (J45–J46.0), atopic dermatitis (L20–L20.9), inflammatory bowel disease (K50–K52, K52.8–K52.9), multiple sclerosis (G35–G35.0), psoriasis (L40–L41.9), and rheumatoid arthritis (M05–M05.9, M08–M09.8).

**Supplementary 2 Detail Information on Mendelian Randomization**

Bidirectional two-sample MR was conducted to investigate the causal relationships between IMID and cancer. Candidate genetic instruments for IMIDs and cancers were identified from the Finngen biobank (*N* = 500,348) and UKBB (*N* = 487,409).

Each instrumental variable (IV) was selected aligning with the foundational causal inference hypothesis. Single nucleotide polymorphisms (SNPs) associated with the exposure of interest (*p*-value < 5 × 10^–6^) were selected^1^. To ensure the independence of SNPs for each exposure (not in linkage disequilibrium), we applied the MR Base function *clump data* to remove correlated SNPs (R^2^ < 0.01, distance = 10,000 kb). We used the 1000 Genomes reference panel to obtain linkage disequilibrium. The strength of the genetic instruments was assessed by calculating the F-statistic. Instruments with an F-statistic >10 were selected and indicating the validity of the selected instruments.

The inverse variance weighted (IVW)^2^ method was used as the primary method to estimate the odds ratio (OR) and 95% confidence interval (CI). Multiple sensitivity analyses such as MR-Egger^3^, Weighted mode^4^, weighted median^5^, and maximum likelihood approaches^6^ were used as complementary analyses to examine the robustness of the results. To test for pleiotropy and heterogeneity, we conducted several sensitivity analyses. The Cochran's Q test was used to assess heterogeneity. Additionally, we performed the MR-Egger intercept test and MR-PRESSO were performed to account for potential violations of the instrumental variable assumptions^7,8^.

**Reference**

1. S. Wang, H. Jiang, H. Qi, D. Luo, T. Qiu, M. Hu “Association Between Periodontitis and Temporomandibular Joint Disorders,” Arthritis Research & Therapy 25, no. 1 (2023): 143, https://doi.org/10.1186/s13075-023-03129-0.

2. S. Burgess, A. Butterworth, S. G. Thompson “Mendelian Randomization Analysis With Multiple Genetic Variants Using Summarized Data,” Genetic Epidemiology 37, no. 7 (2013): 658–665, https://doi.org/10.1002/gepi.21758.

3. J. Bowden, G. D. Smith, S. Burgess “Mendelian Randomization With Invalid Instruments: Effect Estimation and Bias Detection Through Egger Regression,” International Journal of Epidemiology 44, no. 2 (2015): 512–525, https://doi.org/10.1093/ije/dyv080.

4. F. P. Hartwig, G. Davey Smith, J. Bowden “Robust Inference in Summary Data Mendelian Randomization via the Zero Modal Pleiotropy Assumption,” International Journal of Epidemiology 46, no. 6 (2017): 1985–1998, https://doi.org/10.1093/ije/dyx102.

5. J. Bowden, G. Davey Smith, P. C. Haycock, S. Burgess “Consistent Estimation in Mendelian Randomization With Some Invalid Instruments Using a Weighted Median Estimator,” Genetic Epidemiology 40, no. 4 (2016): 304–314, https://doi.org/10.1002/gepi.21965.

6. S. Burgess, F. Dudbridge, S. G. Thompson “Combining Information on Multiple Instrumental Variables in Mendelian Randomization: Comparison of Allele Score and Summarized Data Methods,” Statistics in Medicine 35, no. 11 (2016): 1880–1906, https://doi.org/10.1002/sim.6835.

7. M. Verbanck, C.-Y. Chen, B. Neale, R. Do “Detection of Widespread Horizontal Pleiotropy in Causal Relationships Inferred From Mendelian Randomization Between Complex Traits and Diseases,” Nature Genetics 50, no. 5 (2018): 693–698, https://doi.org/10.1038/s41588-018-0099-7.

8. J. Bowden, F. Del Greco M, C. Minelli, G. Davey Smith, N. Sheehan, J. Thompson “A Framework for the Investigation of Pleiotropy in Two-Sample Summary Data Mendelian Randomization,” Statistics in Medicine 36, no. 11 (2017): 1783–1802, https://doi.org/10.1002/sim.7221.

**Supplementary 3**

**[Supplementary Figure 1.](#_Toc189743254)** [Spearman rank correlations between age-standardized incidence rate of immune-mediated inflammatory diseases and cancer, 2012-2021. 6](#_Toc189743254)

**[Supplementary Figure 2.](#_Toc189743255)** [Spearman rank correlations between age-standardized incidence rate of immune-mediated inflammatory diseases and cancer by healthcare access and quality index. 7](#_Toc189743255)

**[Supplementary Figure 3.](#_Toc189743256)** [Spearman rank correlations between age-standardized incidence rate of immune-mediated inflammatory diseases and cancers among males. 8](#_Toc189743256)

**[Supplementary Figure 4.](#_Toc189743257)** [Spearman rank correlations between age-standardized incidence rate of immune-mediated inflammatory diseases and cancers among females.. 9](#_Toc189743257)

**[Supplementary Figure 5.](#_Toc189743258)** [Spearman rank correlations between age-standardized incidence rate of immune-mediated inflammatory diseases and cancer by age group. 10](#_Toc189743258)

**[Supplementary Figure 6.](#_Toc189743259)** [Hazard Ratios between specific cancers and immune-mediated inflammatory diseases in the UKBB. 11](#_Toc189743259)

**[Supplementary Figure 7.](#_Toc189743260)** [Two-sample Mendelian randomization analysis assessing the causal effect of rheumatoid arthritis on Hodgkin lymphoma.. 12](#_Toc189743260)

**[Supplementary Table 1.](#_Toc211452777)** [Hazard ratios between cancer and immune-mediated inflammatory diseases 14](#_Toc211452777)

**[Supplementary Table 2.](#_Toc211452778)** [Hazard ratios between any immune-mediated inflammatory diseases and cancers 16](#_Toc211452778)

**[Supplementary Table 3.](#_Toc211452779)** [Sensitivity analysis of hazard ratios between any immune-mediated inflammatory diseases and cancers, excluding IMID diagnoses within 1 year after cancer diagnosis 21](#_Toc211452779)

**[Supplementary Table 4.](#_Toc211452780)** [The causal effect between immune-mediated inflammatory diseases and cancers 24](#_Toc211452780)

**[Supplementary Table 5.](#_Toc211452781)** [Causal effect of rheumatoid arthritis on Hodgkin lymphoma 48](#_Toc211452781)


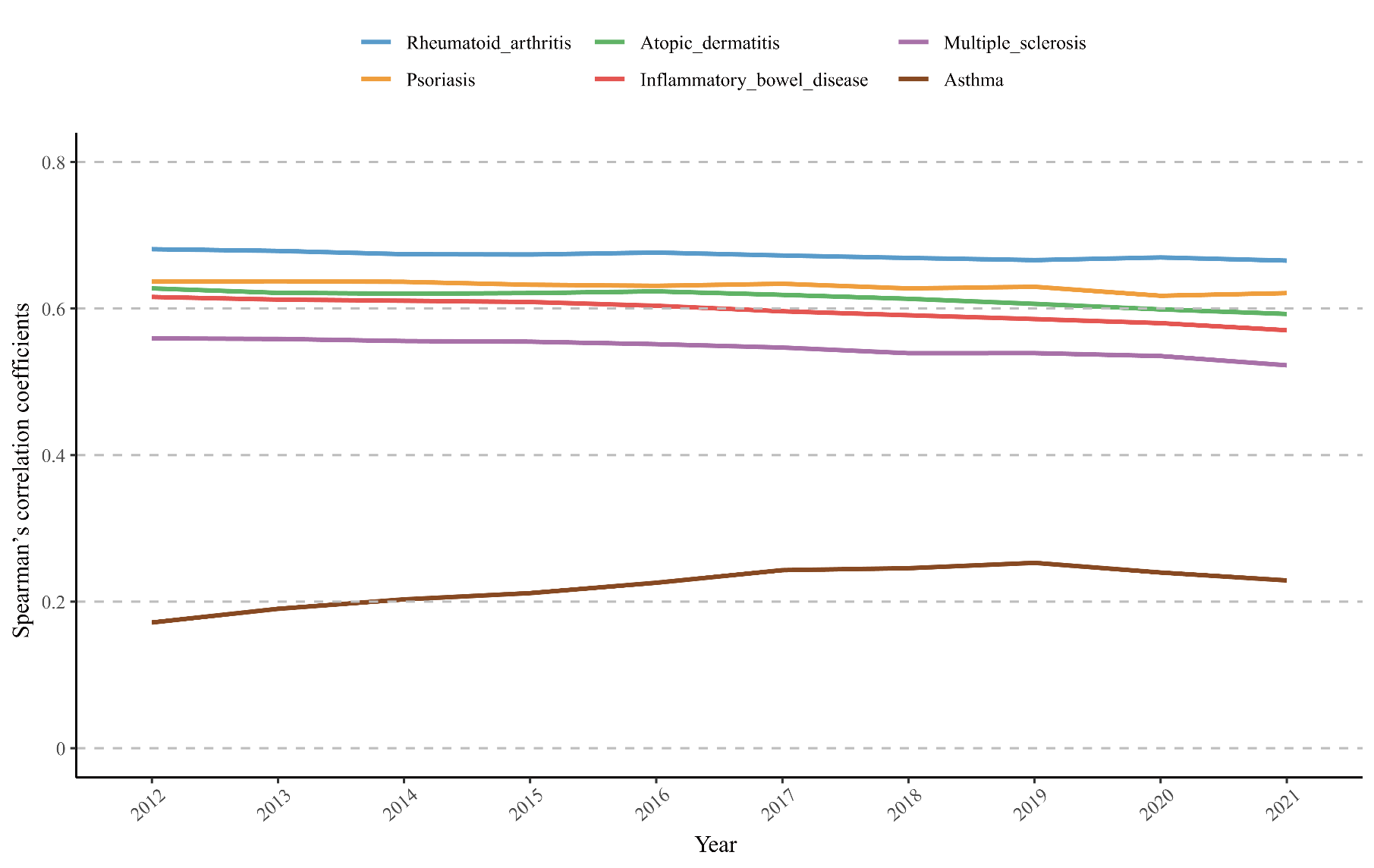


**Supplementary Figure 1.** Spearman rank correlations between age-standardized incidence rate of immune-mediated inflammatory diseases and cancer, 2012–2021.


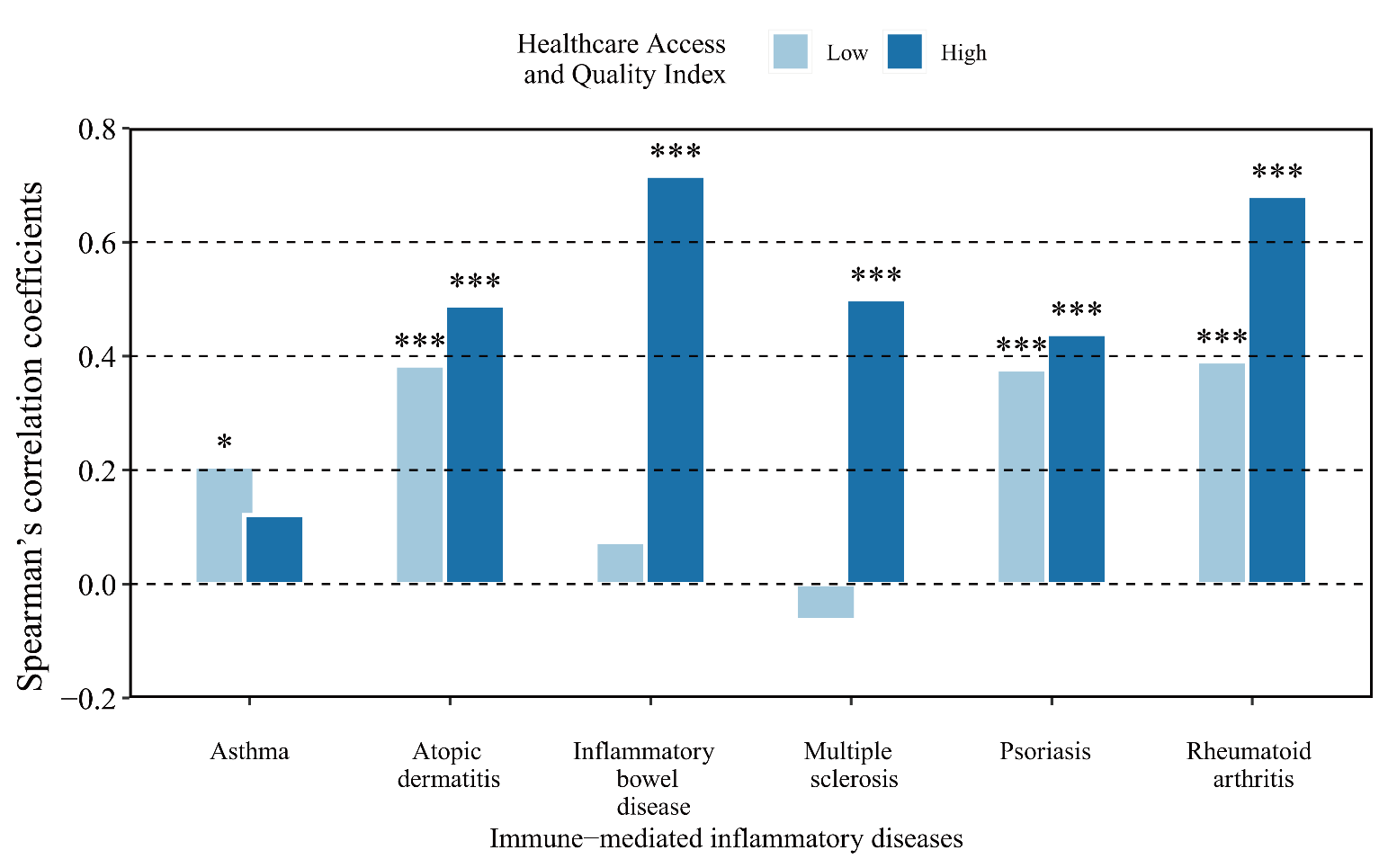


**Supplementary Figure 2.** Spearman rank correlations between age-standardized incidence rate of immune-mediated inflammatory diseases and cancer by healthcare access and quality index. Significance is indicated as follows: *** *p* < 0.001, ** 0.001 ≤ *p* < 0.01, * 0.01 ≤ *p* < 0.05.

**
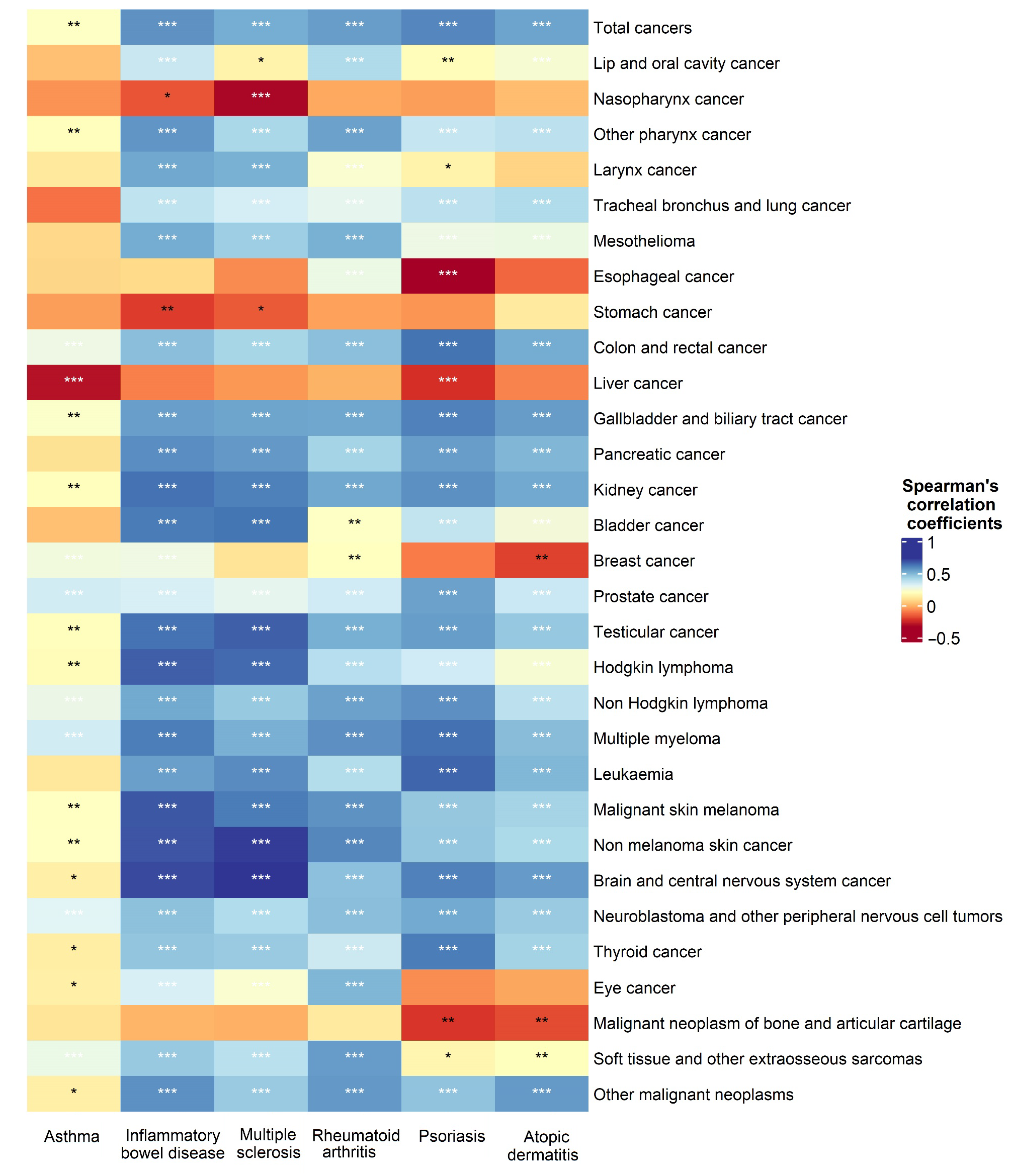
**

**Supplementary Figure 3.** Spearman rank correlations between age-standardized incidence rate of immune-mediated inflammatory diseases and cancers among males. Significance is indicated as follows: *** *p* < 0.001, ** 0.001 ≤ *p* < 0.01, * 0.01 ≤ *p* < 0.05.

**
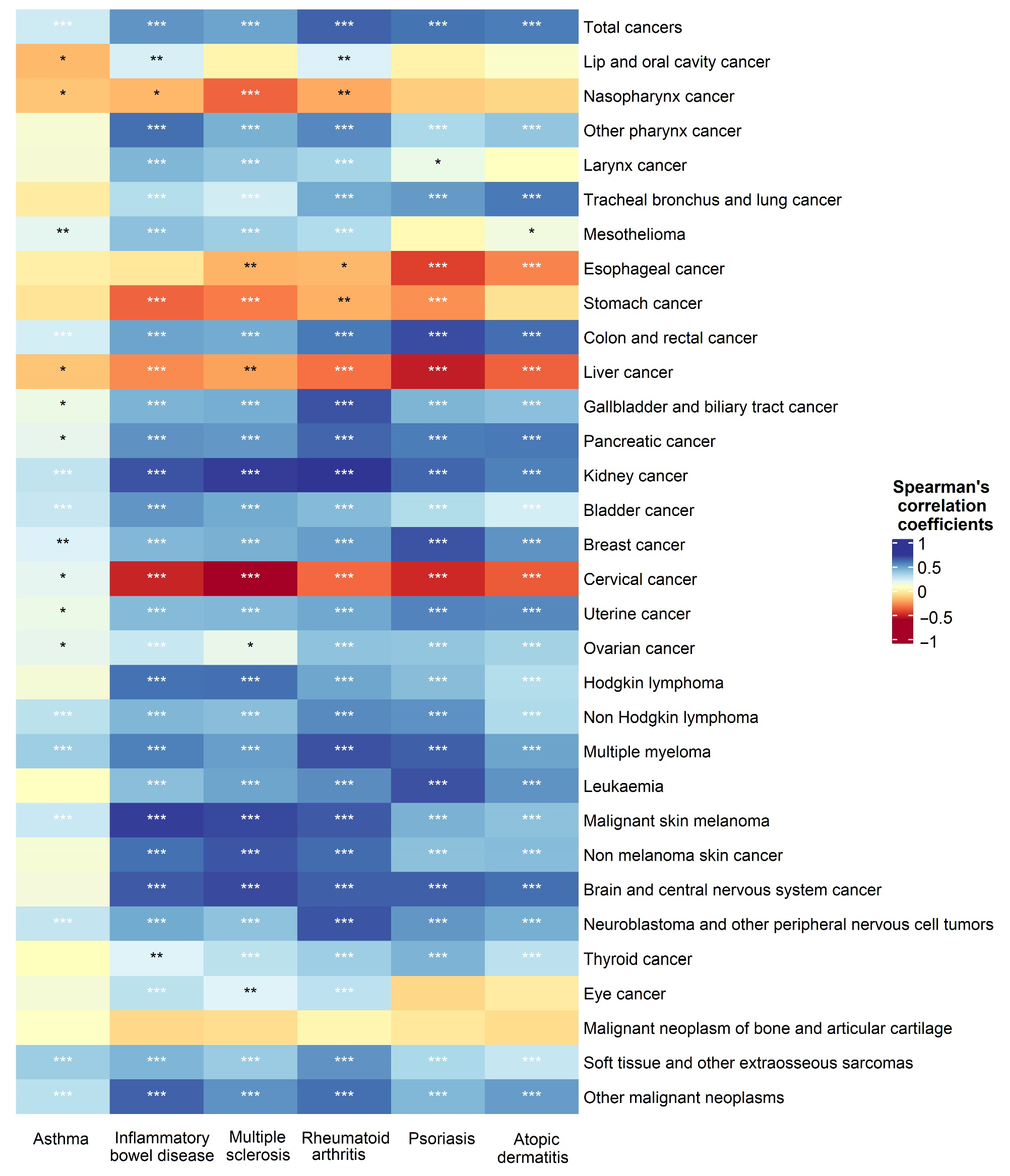
**

**Supplementary Figure 4.** Spearman rank correlations between age-standardized incidence rate of immune-mediated inflammatory diseases and cancers among females. Significance is indicated as follows: *** *p* < 0.001, ** 0.001 ≤ *p* < 0.01, * 0.01 ≤ *p* < 0.05.


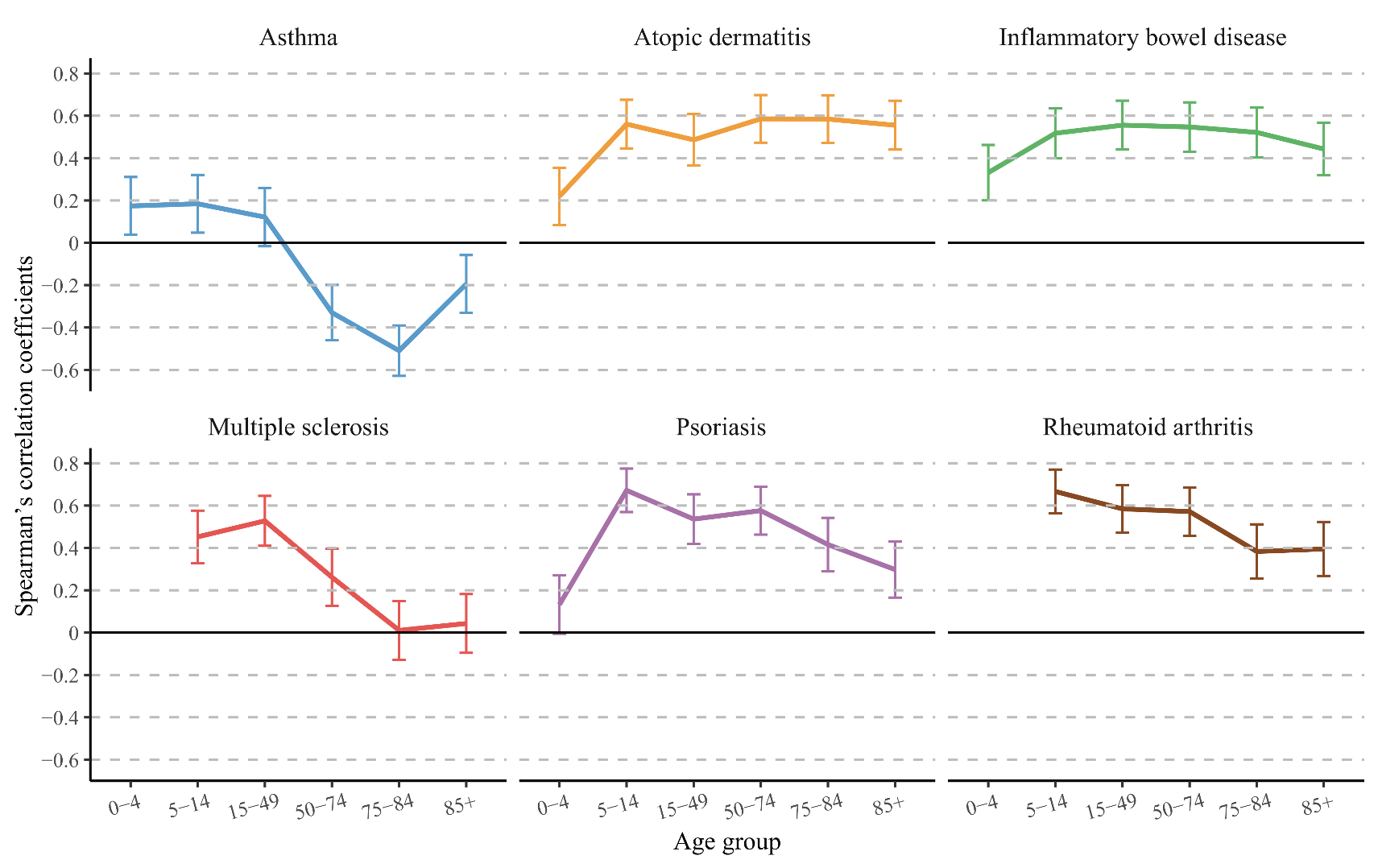


**Supplementary Figure 5.** Spearman rank correlations between age-standardized incidence rate of immune-mediated inflammatory diseases and cancer by age group.

**
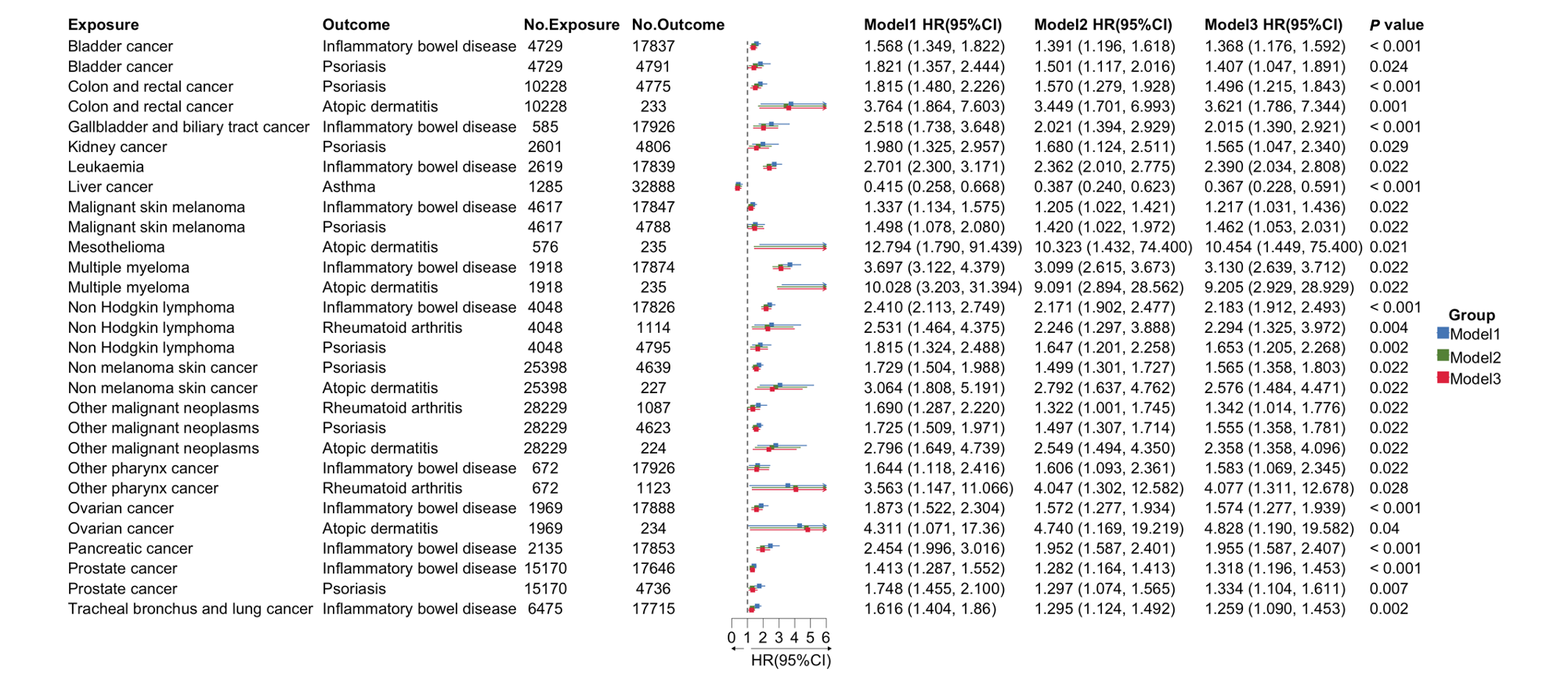
Supplementary Figure 6.** Hazard Ratios between specific cancers and immune-mediated inflammatory diseases in the UKBB. Only significant immune-mediated inflammatory disease and cancer pairs in the Global Burden of Disease and UKBB are shown. Model 1: Unadjusted Cox proportional hazards model; Model 2: Cox proportional hazards model adjusted for age and sex; Model 3: Cox proportional hazards model adjusted for age, sex, BMI, smoking history, alcohol intake, and family history of cancer. CI, confidence interval; HR, hazard ratio; UKBB, UK Biobank. *P* value indicates the maximum *p* value result among model 1, model 2, and model 3.


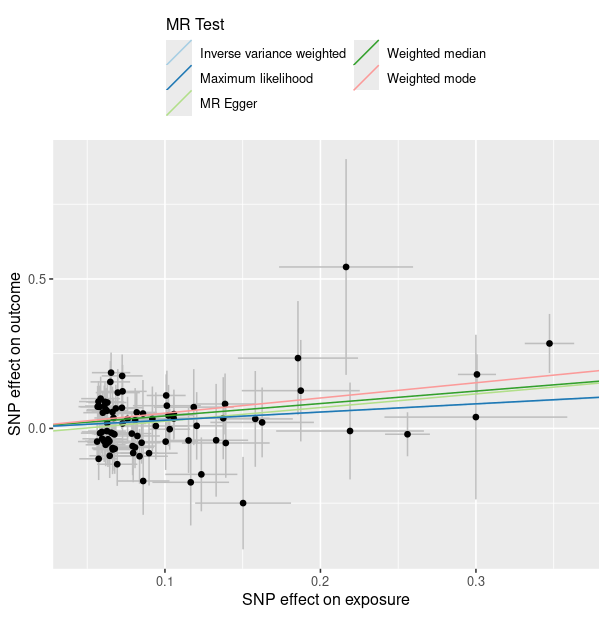

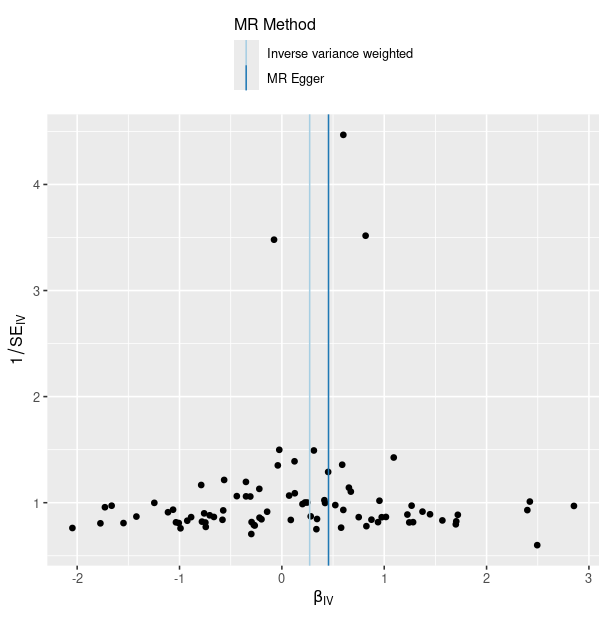


(a) (b)

**Supplementary Figure 7.** Two-sample Mendelian randomization analysis assessing the causal effect of rheumatoid arthritis on Hodgkin lymphoma. (a) Scatter plots of the Mendelian randomization tests. (b) Funnel plot of Mendelian randomization the tests.

**Supplementary Table 1.** Hazard ratios between cancer and immune-mediated inflammatory diseases

| Independent variable | Sample size | Cases | Model 1 ^a^ | | | Model 2 ^b^ | | | Model 3 ^c^ | | |
| --- | --- | --- | --- | --- | --- | --- | --- | --- | --- | --- | --- |
|  |  |  | HR (95%CI) | *P* value | **FDR** | HR (95%CI) | *P* value | **FDR** | HR (95%CI) | *P* value | **FDR** |
| All immune-mediated inflammatory diseases | 81816 | 74579 | 1.117 (1.094, 1.141) | 6.44E-25 | 4.51E-24 | 1.079 (1.057, 1.102) | 1.76E-12 | 1.23E-11 | 1.071 (1.049, 1.095) | 2.74E-10 | 1.92E-09 |
| Asthma | 48432 | 77296 | 1.118 (1.089, 1.148) | 1.63E-16 | 3.81E-16 | 1.048 (1.021, 1.076) | 0.000529 | 0.001233219 | 1.039 (1.011, 1.067) | 0.005456 | 0.012730707 |
| Atopic dermatitis | 299 | 80399 | 1.246 (0.859, 1.806) | 0.246673 | 0.24667312 | 1.310 (0.904, 1.898) | 0.153862 | 0.215406215 | 1.302 (0.893, 1.9) | 0.17025 | 0.238349842 |
| Inflammatory bowel disease | 31405 | 77862 | 1.040 (1.008, 1.073) | 0.01531 | 0.021434513 | 1.042 (1.01, 1.076) | 0.010144 | 0.017752429 | 1.037 (1.005, 1.071) | 0.024457 | 0.04279927 |
| Multiple sclerosis | 2121 | 80369 | 0.709 (0.619, 0.812) | 7.12E-07 | 1.25E-06 | 0.941 (0.822, 1.078) | 0.382524 | 0.446278541 | 0.965 (0.839, 1.11) | 0.616614 | 0.616614127 |
| Psoriasis | 6054 | 79729 | 1.412 (1.312, 1.519) | 2.72E-20 | 9.51E-20 | 1.152 (1.07, 1.239) | 0.000158 | 0.000552818 | 1.129 (1.049, 1.216) | 0.001255 | 0.00439304 |
| Rheumatoid arthritis | 1499 | 80312 | 1.124 (0.964, 1.311) | 0.135943 | 0.158599892 | 0.969 (0.831, 1.13) | 0.688249 | 0.688248873 | 0.951 (0.814, 1.112) | 0.529675 | 0.616614127 |

^a^ Model 1: Unadjusted Cox proportional hazards model; ^b^ Model 2: Cox proportional hazards model adjusted for age and sex; ^c^ Model 3: Cox proportional hazards model adjusted for age, sex, BMI, smoking history, alcohol intake, and family history of cancer. Abbreviations: CI, confidence interval; FDR, False Discovery Rate; HR, hazard ratio.

**Supplementary Table 2.** Hazard ratios between any immune-mediated inflammatory diseases and cancers

| Independent variable | Sample size | Cases | Model 1^a^ | | | Model 2 ^b^ | |  | Model 3 ^c^ | |  |
| --- | --- | --- | --- | --- | --- | --- | --- | --- | --- | --- | --- |
|  |  |  | HR (95%CI) | *P* value | FDR | HR (95%CI) | *P* value | FDR | HR (95%CI) | *P* value | FDR |
| All-site cancer | 98540 | 47723 | 1.126 (1.098, 1.155) | 4.45E-20 | 6.68E-19 | 1.035 (1.008, 1.063) | 0.009623 | 0.027265674 | 1.030 (1.003, 1.057) | 0.027272 | 0.066232572 |
| Lip and oral cavity cancer | 1346 | 54787 | 0.650 (0.510, 0.829) | 0.000522 | 0.001424524 | 0.635 (0.498, 0.810) | 0.000253 | 0.000782689 | 0.657 (0.515, 0.838) | 0.000703 | 0.001992438 |
| Nasopharynx cancer | 84 | 54866 |  |  |  | 0.934 (0.419, 2.079) | 0.86649 | 0.9144637 | 0.985 (0.442, 2.194) | 0.970387 | 0.970387116 |
| Other pharynx cancer | 672 | 54819 | 1.011 (0.761, 1.342) | 0.940021 | 0.94002131 | 1.014 (0.764, 1.347) | 0.921589 | 0.921588991 | 1.044 (0.784, 1.390) | 0.77046 | 0.845020304 |
| Larynx cancer | 466 | 54846 |  |  |  | 0.901 (0.642, 1.262) | 0.543076 | 0.659449767 | 0.908 (0.648, 1.273) | 0.577003 | 0.700646467 |
| Tracheal, bronchus, and lung cancer | 6475 | 54131 | 1.023 (0.922, 1.134) | 0.671421 | 0.774716317 | 0.889 (0.801, 0.986) | 0.026228 | 0.066679482 | 0.878 (0.791, 0.974) | 0.01449 | 0.037896886 |
| Mesothelioma | 576 | 54811 | 0.743 (0.502, 1.099) | 0.137236 | 0.205854702 | 0.651 (0.440, 0.964) | 0.03191 | 0.067808836 | 0.671 (0.454, 0.994) | 0.046644 | 0.105727065 |
| Esophageal cancer | 1970 | 54686 | 1.593 (1.374, 1.846) | 6.73E-10 | 4.04E-09 | 1.455 (1.255, 1.688) | 6.85E-07 | 2.87E-06 | 1.449 (1.249, 1.680) | 9.33E-07 | 3.97E-06 |
| Stomach cancer | 1492 | 54726 | 1.585 (1.341, 1.874) | 6.79E-08 | 2.76E-07 | 1.443 (1.220, 1.706) | 1.79E-05 | 6.10E-05 | 1.430 (1.209, 1.692) | 3.05E-05 | 0.000103638 |
| Colon and rectal cancer | 10228 | 54043 | 1.684 (1.587, 1.787) | 2.70E-66 | 8.10E-65 | 1.553 (1.462, 1.648) | 4.12E-47 | 1.40E-45 | 1.536 (1.446, 1.631) | 3.06E-44 | 1.04E-42 |
| Liver cancer | 1285 | 54703 | 0.945 (0.739, 1.207) | 0.648643 | 0.774716317 | 0.830 (0.649, 1.061) | 0.136961 | 0.221746884 | 0.783 (0.611, 1.002) | 0.05203 | 0.108935305 |
| Gallbladder and biliary tract cancer | 585 | 54795 | 1.062 (0.762, 1.479) | 0.723311 | 0.803678382 | 0.925 (0.664, 1.289) | 0.644332 | 0.755424044 | 0.918 (0.659, 1.279) | 0.611596 | 0.717043627 |
| Pancreatic cancer | 2135 | 54600 | 1.190 (1.001, 1.416) | 0.049187 | 0.09300967 | 1.032 (0.867, 1.228) | 0.723782 | 0.820285833 | 1.027 (0.863, 1.223) | 0.762776 | 0.845020304 |
| Kidney cancer | 2601 | 54605 |  |  |  | 0.930 (0.799, 1.082) | 0.347634 | 0.45459768 | 0.892 (0.766, 1.038) | 0.139877 | 0.211803173 |
| Bladder cancer | 4729 | 54511 | 1.153 (1.042, 1.276) | 0.005945 | 0.012738804 | 1.085 (0.980, 1.202) | 0.11466 | 0.194921508 | 1.060 (0.957, 1.175) | 0.260454 | 0.368976983 |
| Breast cancer | 18334 | 53702 | 0.879 (0.832, 0.928) | 3.51E-06 | 1.17E-05 | 0.806 (0.762, 0.851) | 1.58E-14 | 1.79E-13 | 0.793 (0.750, 0.838) | 2.57E-16 | 4.37E-15 |
| Cervical cancer | 458 | 54852 | 1.127 (0.850, 1.496) | 0.405352 | 0.506690138 | 1.146 (0.864, 1.520) | 0.344808 | 0.45459768 | 1.121 (0.845, 1.488) | 0.427014 | 0.558402597 |
| Uterine cancer | 2580 | 54673 | 0.986 (0.855, 1.138) | 0.848358 | 0.908954839 | 0.853 (0.739, 0.985) | 0.029777 | 0.067493983 | 0.751 (0.650, 0.868) | 0.000106 | 0.0003285 |
| Ovarian cancer | 1969 | 54702 | 1.126 (0.966, 1.313) | 0.130377 | 0.205854702 | 0.989 (0.848, 1.153) | 0.887568 | 0.9144637 | 0.983 (0.842, 1.148) | 0.8271 | 0.854778433 |
| Prostate cancer | 15170 | 53761 | 1.065 (1.000, 1.134) | 0.049605 | 0.09300967 | 1.027 (0.963, 1.095) | 0.422218 | 0.531682547 | 1.053 (0.987, 1.123) | 0.115877 | 0.187610755 |
| Testicular cancer | 371 | 54860 | 0.561 (0.375, 0.840) | 0.004976 | 0.01148229 | 0.705 (0.471, 1.055) | 0.088845 | 0.163350379 | 0.707 (0.472, 1.058) | 0.091884 | 0.156203039 |
| Hodgkin lymphoma | 516 | 54829 | 0.988 (0.732, 1.333) | 0.936807 | 0.94002131 | 1.044 (0.774, 1.409) | 0.775958 | 0.851051205 | 1.033 (0.766, 1.395) | 0.829638 | 0.854778433 |
| Non-Hodgkin lymphoma | 4048 | 54512 | 1.376 (1.245, 1.521) | 4.49E-10 | 3.37E-09 | 1.300 (1.175, 1.437) | 3.10E-07 | 1.51E-06 | 1.301 (1.176, 1.439) | 3.10E-07 | 1.51E-06 |
| Multiple myeloma | 1918 | 54694 | 1.877 (1.634, 2.155) | 5.48E-19 | 5.48E-18 | 1.686 (1.467, 1.937) | 1.65E-13 | 1.40E-12 | 1.674 (1.455, 1.926) | 6.03E-13 | 6.83E-12 |
| Leukaemia | 2619 | 54602 | 1.477 (1.303, 1.675) | 1.11E-09 | 5.53E-09 | 1.373 (1.211, 1.557) | 7.59E-07 | 2.87E-06 | 1.364 (1.202, 1.548) | 1.46E-06 | 5.51E-06 |
| Malignant skin melanoma | 4617 | 54539 |  |  |  | 0.880 (0.786, 0.986) | 0.027456 | 0.066679482 | 0.894 (0.798, 1.002) | 0.054468 | 0.108935305 |
| Non-melanoma skin cancer | 25398 | 53041 | 0.896 (0.851, 0.943) | 2.54E-05 | 7.62E-05 | 0.812 (0.771, 0.856) | 4.46E-15 | 7.59E-14 | 0.833 (0.791, 0.878) | 6.76E-12 | 5.75E-11 |
| Brain and central nervous system cancer | 1353 | 54746 | 0.402 (0.289, 0.560) | 7.35E-08 | 2.76E-07 | 0.376 (0.270, 0.524) | 7.37E-09 | 4.18E-08 | 0.362 (0.258, 0.510) | 5.68E-09 | 3.22E-08 |
| Neuroblastoma and other peripheral nervous cell tumors | 38 | 54869 | 1.541 (0.682, 3.484) | 0.298791 | 0.389727163 | 1.604 (0.709, 3.628) | 0.256265 | 0.363042333 | 1.386 (0.566, 3.392) | 0.475 | 0.598148089 |
| Thyroid cancer | 767 | 54811 | 0.844 (0.642, 1.109) | 0.223226 | 0.318894068 | 0.816 (0.621, 1.072) | 0.144728 | 0.223670629 | 0.816 (0.621, 1.072) | 0.143279 | 0.211803173 |
| Eye cancer | 314 | 54850 | 0.689 (0.441, 1.077) | 0.102478 | 0.170796142 | 0.681 (0.435, 1.064) | 0.091284 | 0.163350379 | 0.665 (0.426, 1.040) | 0.074092 | 0.132586035 |
| Malignant neoplasm of bone and articular cartilage | 283 | 54852 | 0.770 (0.478, 1.240) | 0.281813 | 0.384289835 | 0.759 (0.471, 1.222) | 0.256111 | 0.363042333 | 0.791 (0.491, 1.274) | 0.334817 | 0.45535136 |
| Soft tissue and other extraosseous sarcomas | 711 | 54813 | 0.769 (0.564, 1.050) | 0.09858 | 0.170796142 | 0.745 (0.546, 1.017) | 0.063512 | 0.127024113 | 0.738 (0.539, 1.011) | 0.058577 | 0.110645306 |
| Other malignant neoplasms | 28229 | 52762 | 0.929 (0.885, 0.975) | 0.002792 | 0.006979937 | 0.841 (0.801, 0.883) | 3.92E-12 | 2.67E-11 | 0.861 (0.819, 0.904) | 1.96E-09 | 1.33E-08 |

^a^ Model 1: Unadjusted Cox proportional hazards model; ^b^ Model 2: Cox proportional hazards model adjusted for age and sex; ^c^ Model 3: Cox proportional hazards model adjusted for age, sex, BMI, smoking history, alcohol intake, and family history of cancer. CI, confidence interval; FDR, False Discovery Rate; HR, hazard ratio.

**Supplementary Table 3.** Sensitivity analysis of hazard ratios between any immune-mediated inflammatory diseases and cancers, excluding IMID diagnoses within 1 year after cancer diagnosis

| Independent variable | Sample size | Cases | Model 1^a^ | | | Model 2 ^b^ | | | Model 3 ^c^ | | |
| --- | --- | --- | --- | --- | --- | --- | --- | --- | --- | --- | --- |
|  |  |  | HR (95%CI) | *P* value | FDR | HR (95%CI) | *P* value | FDR | HR (95%CI) | *P* value | FDR |
| All-site cancer | 90355 | 42526 | 0.931 (0.904, 0.958) | 1.66E-06 | 1.03E-05 | 0.869 (0.843, 0.895) | 3.29E-20 | 5.43E-19 | 0.863 (0.837, 0.889) | 1.03E-21 | 1.76E-20 |
| Lip and oral cavity cancer | 1237 | 50349 | 0.488 (0.362, 0.657) | 2.36E-06 | 1.22E-05 | 0.481 (0.357, 0.648) | 1.44E-06 | 5.93E-06 | 0.496 (0.368, 0.668) | 3.98E-06 | 1.35E-05 |
| Nasopharynx cancer | 77 | 50431 |  |  |  | 0.885 (0.368, 2.127) | 0.784502062 | 0.810485251 | 0.931 (0.387, 2.237) | 0.872507781 | 0.872507781 |
| Other pharynx cancer | 621 | 50376 | 0.624 (0.424, 0.916) | 0.01617044 | 0.031330228 | 0.63 (0.429, 0.925) | 0.018547147 | 0.032213465 | 0.635 (0.429, 0.941) | 0.023570074 | 0.042178028 |
| Larynx cancer | 433 | 50409 | 0.813 (0.557, 1.188) | 0.285379152 | 0.353870148 | 0.808 (0.553, 1.181) | 0.270850286 | 0.343771517 | 0.814 (0.557, 1.189) | 0.286196727 | 0.360395879 |
| Tracheal, bronchus, and lung cancer | 5669 | 49606 | 0.561 (0.482, 0.652) | 6.62E-14 | 1.03E-12 | 0.492 (0.423, 0.573) | 5.11E-20 | 5.62E-19 | 0.486 (0.417, 0.566) | 1.87E-20 | 2.12E-19 |
| Mesothelioma | 523 | 50376 | 0.384 (0.213, 0.694) | 0.00151593 | 0.004699382 | 0.339 (0.188, 0.613) | 0.000338922 | 0.001016766 | 0.349 (0.193, 0.631) | 0.000485825 | 0.001376505 |
| Esophageal cancer | 1674 | 50177 | 0.717 (0.565, 0.909) | 0.006081617 | 0.015710843 | 0.661 (0.521, 0.839) | 0.000671235 | 0.001703905 | 0.659 (0.519, 0.836) | 0.000591323 | 0.001546537 |
| Stomach cancer | 1281 | 50232 | 0.642 (0.484, 0.852) | 0.002181288 | 0.006147265 | 0.59 (0.444, 0.783) | 0.000264167 | 0.00087175 | 0.59 (0.444, 0.783) | 0.00026133 | 0.000807748 |
| Colon and rectum cancer | 9020 | 49228 | 1.012 (0.933, 1.098) | 0.775061255 | 0.80089663 | 0.944 (0.869, 1.024) | 0.165268513 | 0.218154437 | 0.928 (0.854, 1.008) | 0.076603408 | 0.118387085 |
| Liver cancer | 1147 | 50264 | 0.476 (0.326, 0.694) | 0.000114693 | 0.000444436 | 0.422 (0.29, 0.616) | 7.61E-06 | 2.79E-05 | 0.404 (0.277, 0.59) | 2.60E-06 | 9.83E-06 |
| Gallbladder and biliary tract cancer | 514 | 50354 | 0.536 (0.323, 0.889) | 0.015701445 | 0.031330228 | 0.471 (0.284, 0.782) | 0.003593304 | 0.007411189 | 0.468 (0.282, 0.777) | 0.00331354 | 0.007510691 |
| Pancreatic cancer | 1822 | 50105 | 0.435 (0.318, 0.595) | 2.01E-07 | 1.56E-06 | 0.381 (0.278, 0.521) | 1.73E-09 | 8.16E-09 | 0.382 (0.279, 0.523) | 1.96E-09 | 9.53E-09 |
| Kidney cancer | 2362 | 50166 | 0.863 (0.724, 1.027) | 0.097690632 | 0.137654981 | 0.807 (0.678, 0.962) | 0.016425114 | 0.030112709 | 0.771 (0.647, 0.92) | 0.003787615 | 0.008048682 |
| Bladder cancer | 4296 | 50052 | 0.996 (0.886, 1.119) | 0.944984306 | 0.944984306 | 0.947 (0.842, 1.064) | 0.357190986 | 0.436566761 | 0.921 (0.819, 1.036) | 0.170851756 | 0.232358388 |
| Breast cancer | 17211 | 49246 | 0.782 (0.736, 0.832) | 3.46E-15 | 1.07E-13 | 0.721 (0.678, 0.767) | 2.59E-25 | 8.54E-24 | 0.709 (0.667, 0.755) | 1.97E-27 | 6.70E-26 |
| Cervical cancer | 429 | 50410 |  |  |  | 0.956 (0.691, 1.323) | 0.785925092 | 0.810485251 | 0.934 (0.674, 1.293) | 0.679897141 | 0.722390712 |
| Uterine cancer | 2385 | 50230 | 0.864 (0.734, 1.016) | 0.07675742 | 0.118974001 | 0.752 (0.64, 0.885) | 0.000610499 | 0.001678872 | 0.66 (0.56, 0.778) | 7.55E-07 | 3.21E-06 |
| Ovarian cancer | 1809 | 50246 | 0.831 (0.687, 1.005) | 0.056353234 | 0.09194475 | 0.736 (0.609, 0.89) | 0.001560898 | 0.003679258 | 0.725 (0.599, 0.879) | 0.001033781 | 0.002510611 |
| Prostate cancer | 13685 | 49271 | 0.972 (0.906, 1.043) | 0.430587781 | 0.494378563 | 0.945 (0.879, 1.016) | 0.124584855 | 0.178752183 | 0.967 (0.899, 1.04) | 0.362174761 | 0.424618685 |
| Testicular cancer | 365 | 50425 | 0.558 (0.366, 0.851) | 0.006679749 | 0.015928631 | 0.701 (0.46, 1.069) | 0.098983234 | 0.155545081 | 0.703 (0.461, 1.072) | 0.101325867 | 0.143544978 |
| Hodgkin lymphoma | 481 | 50395 | 0.845 (0.601, 1.19) | 0.335347537 | 0.399837448 | 0.902 (0.641, 1.269) | 0.55348982 | 0.629833243 | 0.891 (0.633, 1.254) | 0.508809308 | 0.576650549 |
| Non-Hodgkin lymphoma | 3671 | 50032 | 1.082 (0.959, 1.221) | 0.19784799 | 0.255553653 | 1.033 (0.916, 1.165) | 0.596986393 | 0.656685032 | 1.033 (0.915, 1.166) | 0.599794527 | 0.657839158 |
| Multiple myeloma | 1664 | 50204 | 1.257 (1.046, 1.51) | 0.014692843 | 0.031330228 | 1.141 (0.95, 1.371) | 0.158898897 | 0.218154437 | 1.134 (0.942, 1.365) | 0.183358901 | 0.239777025 |
| Leukemia | 2330 | 50127 | 1.047 (0.892, 1.228) | 0.576765257 | 0.616542171 | 0.984 (0.838, 1.154) | 0.840445767 | 0.840445767 | 0.975 (0.83, 1.145) | 0.759442278 | 0.78245568 |
| Malignant skin melanoma | 4189 | 50097 | 0.869 (0.767, 0.986) | 0.028912743 | 0.049794168 | 0.825 (0.728, 0.935) | 0.002690628 | 0.005919381 | 0.836 (0.737, 0.949) | 0.00540694 | 0.010813879 |
| Non-melanoma skin cancer | 23462 | 48664 | 0.891 (0.843, 0.941) | 3.99E-05 | 0.000176753 | 0.816 (0.771, 0.862) | 7.52E-13 | 6.21E-12 | 0.834 (0.789, 0.882) | 2.18E-10 | 1.48E-09 |
| Brain and central nervous system cancer | 1235 | 50309 | 0.199 (0.12, 0.331) | 4.22E-10 | 4.36E-09 | 0.188 (0.113, 0.312) | 9.69E-11 | 5.33E-10 | 0.179 (0.106, 0.302) | 1.24E-10 | 1.06E-09 |
| Neuroblastoma and other peripheral nervous cell tumors | 36 | 50434 | 1.399 (0.572, 3.425) | 0.461732102 | 0.511203399 | 1.462 (0.597, 3.579) | 0.405914676 | 0.47839944 | 1.521 (0.62, 3.73) | 0.359925815 | 0.424618685 |
| Thyroid cancer | 722 | 50374 | 0.766 (0.565, 1.038) | 0.085325885 | 0.125957259 | 0.746 (0.551, 1.01) | 0.058301121 | 0.096196849 | 0.746 (0.55, 1.01) | 0.05824446 | 0.099015583 |
| Eye cancer | 294 | 50415 | 0.686 (0.428, 1.099) | 0.117128024 | 0.157868207 | 0.681 (0.425, 1.092) | 0.110658042 | 0.165987063 | 0.666 (0.415, 1.068) | 0.091530262 | 0.135305604 |
| Malignant neoplasm of bone and articular cartilage | 262 | 50413 |  |  |  |  |  |  | 0.581 (0.321, 1.049) | 0.07174671 | 0.11616134 |
| Soft tissue and other extraosseous sarcomas | 648 | 50377 | 0.655 (0.457, 0.938) | 0.020802714 | 0.037934361 | 0.64 (0.447, 0.917) | 0.014855835 | 0.028837797 | 0.628 (0.436, 0.905) | 0.012587749 | 0.023776859 |
| Other malignant neoplasms | 26036 | 48369 | 0.904 (0.858, 0.953) | 0.000156158 | 0.000537878 | 0.826 (0.784, 0.871) | 1.48E-12 | 9.78E-12 | 0.843 (0.799, 0.889) | 2.85E-10 | 1.61E-09 |

^a^ Model 1: Unadjusted Cox proportional hazards model; ^b^ Model 2: Cox proportional hazards model adjusted for age and sex; ^c^ Model 3: Cox proportional hazards model adjusted for age, sex, BMI, smoking history, alcohol intake, and family history of cancer. CI, confidence interval; FDR, False Discovery Rate; HR, hazard ratio.

**Supplementary Table 4.** The causal effect between immune-mediated inflammatory diseases and cancers

| **Expose** | **Outcome** | **MR Methods** | **OR** | **OR.lower** | **OR.upper** | ***P*-Value** | **nSNP** | **Heterogeneity** | **Horizontal pleiotropy** |
| --- | --- | --- | --- | --- | --- | --- | --- | --- | --- |
| Asthma | Hodgkin lymphoma | IVW | 1.158 | 0.885 | 1.515 | 0.285 | 142 | 0.290 | 0.533 |
| Asthma | Hodgkin lymphoma | MR Egger | 0.937 | 0.458 | 1.918 | 0.859 | 142 |  |  |
| Asthma | Hodgkin lymphoma | Weighted median | 0.977 | 0.638 | 1.495 | 0.914 | 142 |  |  |
| Asthma | Hodgkin lymphoma | Weighted mode | 0.905 | 0.439 | 1.865 | 0.787 | 142 |  |  |
| Asthma | Hodgkin lymphoma | Maximum likelihood | 1.160 | 0.890 | 1.513 | 0.272 | 142 |  |  |
| Asthma | Non-Hodgkin lymphoma | IVW | 1.022 | 0.925 | 1.128 | 0.674 | 133 | 0.427 | 0.087 |
| Asthma | Non-Hodgkin lymphoma | MR Egger | 0.823 | 0.632 | 1.073 | 0.152 | 133 |  |  |
| Asthma | Non-Hodgkin lymphoma | Weighted median | 0.927 | 0.796 | 1.080 | 0.331 | 133 |  |  |
| Asthma | Non-Hodgkin lymphoma | Weighted mode | 0.777 | 0.571 | 1.058 | 0.112 | 133 |  |  |
| Asthma | Non-Hodgkin lymphoma | Maximum likelihood | 1.022 | 0.925 | 1.130 | 0.671 | 133 |  |  |
| Atopic dermatitis | Ovarian cancer | IVW | 0.990 | 0.971 | 1.009 | 0.301 | 42 | 0.055 | 0.555 |
| Atopic dermatitis | Ovarian cancer | MR Egger | 0.980 | 0.944 | 1.018 | 0.303 | 42 |  |  |
| Atopic dermatitis | Ovarian cancer | Weighted median | 0.986 | 0.964 | 1.007 | 0.187 | 42 |  |  |
| Atopic dermatitis | Ovarian cancer | Weighted mode | 0.982 | 0.964 | 1.001 | 0.071 | 42 |  |  |
| Atopic dermatitis | Ovarian cancer | Maximum likelihood | 0.990 | 0.973 | 1.006 | 0.225 | 42 |  |  |
| Atopic dermatitis | Hodgkin lymphoma | IVW | 1.103 | 0.871 | 1.396 | 0.415 | 103 | 0.319 | 0.847 |
| Atopic dermatitis | Hodgkin lymphoma | MR Egger | 1.046 | 0.581 | 1.884 | 0.882 | 103 |  |  |
| Atopic dermatitis | Hodgkin lymphoma | Weighted median | 1.151 | 0.801 | 1.653 | 0.447 | 103 |  |  |
| Atopic dermatitis | Hodgkin lymphoma | Weighted mode | 2.056 | 0.699 | 6.044 | 0.193 | 103 |  |  |
| Atopic dermatitis | Hodgkin lymphoma | Maximum likelihood | 1.105 | 0.876 | 1.394 | 0.400 | 103 |  |  |
| Atopic dermatitis | Non-Hodgkin lymphoma | IVW | 1.010 | 0.931 | 1.097 | 0.806 | 107 | 0.385 | 0.172 |
| Atopic dermatitis | Non-Hodgkin lymphoma | MR Egger | 0.887 | 0.724 | 1.086 | 0.249 | 107 |  |  |
| Atopic dermatitis | Non-Hodgkin lymphoma | Weighted median | 0.985 | 0.873 | 1.112 | 0.808 | 107 |  |  |
| Atopic dermatitis | Non-Hodgkin lymphoma | Weighted mode | 0.954 | 0.772 | 1.179 | 0.665 | 107 |  |  |
| Atopic dermatitis | Non-Hodgkin lymphoma | Maximum likelihood | 1.011 | 0.931 | 1.096 | 0.800 | 107 |  |  |
| Inflammatory bowel disease | Tracheal, bronchus, and lung cancer | IVW | 0.978 | 0.919 | 1.040 | 0.474 | 14 | 0.584 | 0.106 |
| Inflammatory bowel disease | Tracheal, bronchus, and lung cancer | MR Egger | 0.951 | 0.888 | 1.019 | 0.181 | 14 |  |  |
| Inflammatory bowel disease | Tracheal, bronchus, and lung cancer | Weighted median | 0.970 | 0.899 | 1.046 | 0.427 | 14 |  |  |
| Inflammatory bowel disease | Tracheal, bronchus, and lung cancer | Weighted mode | 0.966 | 0.897 | 1.040 | 0.378 | 14 |  |  |
| Inflammatory bowel disease | Tracheal, bronchus, and lung cancer | Maximum likelihood | 0.978 | 0.919 | 1.039 | 0.469 | 14 |  |  |
| Inflammatory bowel disease | Pancreatic cancer | IVW | 0.993 | 0.890 | 1.106 | 0.893 | 18 | 0.362 | 0.239 |
| Inflammatory bowel disease | Pancreatic cancer | MR Egger | 1.028 | 0.911 | 1.160 | 0.660 | 18 |  |  |
| Inflammatory bowel disease | Pancreatic cancer | Weighted median | 1.008 | 0.887 | 1.145 | 0.908 | 18 |  |  |
| Inflammatory bowel disease | Pancreatic cancer | Weighted mode | 1.016 | 0.902 | 1.144 | 0.794 | 18 |  |  |
| Inflammatory bowel disease | Pancreatic cancer | Maximum likelihood | 0.993 | 0.894 | 1.103 | 0.890 | 18 |  |  |
| Inflammatory bowel disease | Kidney cancer | IVW | 1.032 | 0.939 | 1.135 | 0.508 | 18 | 0.514 | 0.829 |
| Inflammatory bowel disease | Kidney cancer | MR Egger | 1.038 | 0.933 | 1.156 | 0.503 | 18 |  |  |
| Inflammatory bowel disease | Kidney cancer | Weighted median | 1.046 | 0.930 | 1.176 | 0.456 | 18 |  |  |
| Inflammatory bowel disease | Kidney cancer | Weighted mode | 1.046 | 0.941 | 1.162 | 0.417 | 18 |  |  |
| Inflammatory bowel disease | Kidney cancer | Maximum likelihood | 1.032 | 0.939 | 1.135 | 0.508 | 18 |  |  |
| Inflammatory bowel disease | Non-Hodgkin lymphoma | IVW | 0.982 | 0.908 | 1.063 | 0.657 | 18 | 0.388 | 0.105 |
| Inflammatory bowel disease | Non-Hodgkin lymphoma | MR Egger | 1.018 | 0.934 | 1.109 | 0.695 | 18 |  |  |
| Inflammatory bowel disease | Non-Hodgkin lymphoma | Weighted median | 0.995 | 0.909 | 1.089 | 0.910 | 18 |  |  |
| Inflammatory bowel disease | Non-Hodgkin lymphoma | Weighted mode | 0.995 | 0.916 | 1.081 | 0.907 | 18 |  |  |
| Inflammatory bowel disease | Non-Hodgkin lymphoma | Maximum likelihood | 0.982 | 0.909 | 1.061 | 0.652 | 18 |  |  |
| Inflammatory bowel disease | Multiple myeloma | IVW | 1.009 | 0.903 | 1.126 | 0.880 | 18 | 0.566 | 0.289 |
| Inflammatory bowel disease | Multiple myeloma | MR Egger | 1.042 | 0.920 | 1.181 | 0.526 | 18 |  |  |
| Inflammatory bowel disease | Multiple myeloma | Weighted median | 1.072 | 0.929 | 1.238 | 0.339 | 18 |  |  |
| Inflammatory bowel disease | Multiple myeloma | Weighted mode | 1.072 | 0.945 | 1.217 | 0.296 | 18 |  |  |
| Inflammatory bowel disease | Multiple myeloma | Maximum likelihood | 1.009 | 0.902 | 1.129 | 0.880 | 18 |  |  |
| Inflammatory bowel disease | Leukaemia | IVW | 1.001 | 0.912 | 1.099 | 0.988 | 18 | 0.729 | 0.074 |
| Inflammatory bowel disease | Leukaemia | MR Egger | 1.050 | 0.945 | 1.167 | 0.378 | 18 |  |  |
| Inflammatory bowel disease | Leukaemia | Weighted median | 1.021 | 0.903 | 1.155 | 0.739 | 18 |  |  |
| Inflammatory bowel disease | Leukaemia | Weighted mode | 1.021 | 0.923 | 1.130 | 0.692 | 18 |  |  |
| Inflammatory bowel disease | Leukaemia | Maximum likelihood | 1.001 | 0.911 | 1.099 | 0.988 | 18 |  |  |
| Psoriasis | Tracheal, bronchus, and lung cancer | IVW | 0.985 | 0.931 | 1.041 | 0.585 | 67 | 0.003 | 0.179 |
| Psoriasis | Tracheal, bronchus, and lung cancer | MR Egger | 1.035 | 0.945 | 1.134 | 0.461 | 67 |  |  |
| Psoriasis | Tracheal, bronchus, and lung cancer | Weighted median | 1.086 | 1.010 | 1.168 | 0.025 | 67 |  |  |
| Psoriasis | Tracheal, bronchus, and lung cancer | Weighted mode | 1.093 | 1.007 | 1.185 | 0.037 | 67 |  |  |
| Psoriasis | Tracheal, bronchus, and lung cancer | Maximum likelihood | 0.984 | 0.940 | 1.030 | 0.494 | 67 |  |  |
| Psoriasis | Hodgkin lymphoma | IVW | 0.919 | 0.766 | 1.104 | 0.368 | 70 | 0.036 | 0.193 |
| Psoriasis | Hodgkin lymphoma | MR Egger | 0.781 | 0.577 | 1.058 | 0.115 | 70 |  |  |
| Psoriasis | Hodgkin lymphoma | Weighted median | 0.856 | 0.647 | 1.133 | 0.277 | 70 |  |  |
| Psoriasis | Hodgkin lymphoma | Weighted mode | 0.821 | 0.641 | 1.051 | 0.122 | 70 |  |  |
| Psoriasis | Hodgkin lymphoma | Maximum likelihood | 0.919 | 0.782 | 1.079 | 0.301 | 70 |  |  |
| Psoriasis | Non-Hodgkin lymphoma | IVW | 0.993 | 0.934 | 1.056 | 0.830 | 65 | 0.340 | 0.459 |
| Psoriasis | Non-Hodgkin lymphoma | MR Egger | 0.964 | 0.872 | 1.066 | 0.475 | 65 |  |  |
| Psoriasis | Non-Hodgkin lymphoma | Weighted median | 1.002 | 0.906 | 1.109 | 0.969 | 65 |  |  |
| Psoriasis | Non-Hodgkin lymphoma | Weighted mode | 0.990 | 0.902 | 1.086 | 0.826 | 65 |  |  |
| Psoriasis | Non-Hodgkin lymphoma | Maximum likelihood | 0.993 | 0.935 | 1.055 | 0.828 | 65 |  |  |
| Psoriasis | Neuroblastoma and other peripheral nervous cell tumors | IVW | 0.493 | 0.271 | 0.896 | 0.020 | 70 | 0.816 | 0.801 |
| Psoriasis | Neuroblastoma and other peripheral nervous cell tumors | MR Egger | 0.443 | 0.161 | 1.220 | 0.120 | 70 |  |  |
| Psoriasis | Neuroblastoma and other peripheral nervous cell tumors | Weighted median | 0.341 | 0.129 | 0.903 | 0.030 | 70 |  |  |
| Psoriasis | Neuroblastoma and other peripheral nervous cell tumors | Weighted mode | 0.375 | 0.147 | 0.960 | 0.045 | 70 |  |  |
| Psoriasis | Neuroblastoma and other peripheral nervous cell tumors | Maximum likelihood | 0.489 | 0.268 | 0.894 | 0.020 | 70 |  |  |
| Rheumatoid arthritis | Other pharynx cancer | IVW | 1.061 | 0.900 | 1.251 | 0.483 | 85 | 0.339 | 0.092 |
| Rheumatoid arthritis | Other pharynx cancer | MR Egger | 1.358 | 0.978 | 1.884 | 0.071 | 85 |  |  |
| Rheumatoid arthritis | Other pharynx cancer | Weighted median | 1.334 | 1.022 | 1.742 | 0.034 | 85 |  |  |
| Rheumatoid arthritis | Other pharynx cancer | Weighted mode | 1.215 | 0.915 | 1.614 | 0.181 | 85 |  |  |
| Rheumatoid arthritis | Other pharynx cancer | Maximum likelihood | 1.062 | 0.903 | 1.249 | 0.467 | 85 |  |  |
| Rheumatoid arthritis | Tracheal, bronchus, and lung cancer | IVW | 1.041 | 0.981 | 1.104 | 0.186 | 79 | 0.407 | 0.322 |
| Rheumatoid arthritis | Tracheal, bronchus, and lung cancer | MR Egger | 1.105 | 0.969 | 1.260 | 0.142 | 79 |  |  |
| Rheumatoid arthritis | Tracheal, bronchus, and lung cancer | Weighted median | 1.116 | 1.013 | 1.229 | 0.027 | 79 |  |  |
| Rheumatoid arthritis | Tracheal, bronchus, and lung cancer | Weighted mode | 1.118 | 0.997 | 1.252 | 0.059 | 79 |  |  |
| Rheumatoid arthritis | Tracheal, bronchus, and lung cancer | Maximum likelihood | 1.042 | 0.982 | 1.105 | 0.177 | 79 |  |  |
| Rheumatoid arthritis | Hodgkin lymphoma | IVW | 1.309 | 1.094 | 1.567 | 0.003 | 85 | 0.512 | 0.245 |
| Rheumatoid arthritis | Hodgkin lymphoma | MR Egger | 1.570 | 1.103 | 2.236 | 0.014 | 85 |  |  |
| Rheumatoid arthritis | Hodgkin lymphoma | Weighted median | 1.516 | 1.123 | 2.048 | 0.007 | 85 |  |  |
| Rheumatoid arthritis | Hodgkin lymphoma | Weighted mode | 1.601 | 1.129 | 2.269 | 0.010 | 85 |  |  |
| Rheumatoid arthritis | Hodgkin lymphoma | Maximum likelihood | 1.313 | 1.095 | 1.575 | 0.003 | 85 |  |  |
| Rheumatoid arthritis | Non-Hodgkin lymphoma | IVW | 1.039 | 0.972 | 1.110 | 0.266 | 80 | 0.432 | 0.253 |
| Rheumatoid arthritis | Non-Hodgkin lymphoma | MR Egger | 1.111 | 0.973 | 1.268 | 0.124 | 80 |  |  |
| Rheumatoid arthritis | Non-Hodgkin lymphoma | Weighted median | 1.108 | 0.987 | 1.244 | 0.082 | 80 |  |  |
| Rheumatoid arthritis | Non-Hodgkin lymphoma | Weighted mode | 1.119 | 0.989 | 1.266 | 0.079 | 80 |  |  |
| Rheumatoid arthritis | Non-Hodgkin lymphoma | Maximum likelihood | 1.039 | 0.972 | 1.111 | 0.259 | 80 |  |  |
| Rheumatoid arthritis | Multiple myeloma | IVW | 1.129 | 1.010 | 1.262 | 0.033 | 84 | 0.129 | 0.552 |
| Rheumatoid arthritis | Multiple myeloma | MR Egger | 1.209 | 0.940 | 1.556 | 0.143 | 84 |  |  |
| Rheumatoid arthritis | Multiple myeloma | Weighted median | 1.179 | 0.973 | 1.428 | 0.093 | 84 |  |  |
| Rheumatoid arthritis | Multiple myeloma | Weighted mode | 1.170 | 0.958 | 1.429 | 0.128 | 84 |  |  |
| Rheumatoid arthritis | Multiple myeloma | Maximum likelihood | 1.132 | 1.019 | 1.257 | 0.021 | 84 |  |  |
| Bladder cancer | Inflammatory bowel disease | IVW | 1.002 | 0.992 | 1.013 | 0.671 | 25 | 0.099 | 0.526 |
| Bladder cancer | Inflammatory bowel disease | MR Egger | 1.001 | 0.989 | 1.013 | 0.917 | 25 |  |  |
| Bladder cancer | Inflammatory bowel disease | Weighted median | 1.002 | 0.992 | 1.013 | 0.689 | 25 |  |  |
| Bladder cancer | Inflammatory bowel disease | Weighted mode | 1.001 | 0.992 | 1.011 | 0.771 | 25 |  |  |
| Bladder cancer | Inflammatory bowel disease | Maximum likelihood | 1.002 | 0.993 | 1.012 | 0.619 | 25 |  |  |
| Bladder cancer | Psoriasis | IVW | 1.004 | 0.989 | 1.019 | 0.607 | 25 | 0.000 | 0.491 |
| Bladder cancer | Psoriasis | MR Egger | 1.001 | 0.985 | 1.018 | 0.873 | 25 |  |  |
| Bladder cancer | Psoriasis | Weighted median | 1.002 | 0.992 | 1.013 | 0.661 | 25 |  |  |
| Bladder cancer | Psoriasis | Weighted mode | 1.002 | 0.993 | 1.012 | 0.671 | 25 |  |  |
| Bladder cancer | Psoriasis | Maximum likelihood | 1.004 | 0.994 | 1.014 | 0.420 | 25 |  |  |
| Colon and rectal cancer | Atopic dermatitis | IVW | 0.998 | 0.965 | 1.032 | 0.905 | 44 | 0.187 | 0.926 |
| Colon and rectal cancer | Atopic dermatitis | MR Egger | 0.994 | 0.904 | 1.093 | 0.898 | 44 |  |  |
| Colon and rectal cancer | Atopic dermatitis | Weighted median | 0.985 | 0.941 | 1.031 | 0.510 | 44 |  |  |
| Colon and rectal cancer | Atopic dermatitis | Weighted mode | 0.969 | 0.902 | 1.041 | 0.394 | 44 |  |  |
| Colon and rectal cancer | Atopic dermatitis | Maximum likelihood | 0.998 | 0.967 | 1.030 | 0.896 | 44 |  |  |
| Colon and rectal cancer | Psoriasis | IVW | 1.007 | 0.957 | 1.060 | 0.786 | 44 | 0.136 | 0.597 |
| Colon and rectal cancer | Psoriasis | MR Egger | 0.971 | 0.842 | 1.120 | 0.691 | 44 |  |  |
| Colon and rectal cancer | Psoriasis | Weighted median | 0.985 | 0.919 | 1.056 | 0.674 | 44 |  |  |
| Colon and rectal cancer | Psoriasis | Weighted mode | 0.929 | 0.827 | 1.043 | 0.220 | 44 |  |  |
| Colon and rectal cancer | Psoriasis | Maximum likelihood | 1.007 | 0.961 | 1.055 | 0.759 | 44 |  |  |
| Gallbladder and biliary tract cancer | Inflammatory bowel disease | IVW | 0.997 | 0.981 | 1.012 | 0.671 | 29 | 0.605 | 0.738 |
| Gallbladder and biliary tract cancer | Inflammatory bowel disease | MR Egger | 0.993 | 0.966 | 1.020 | 0.607 | 29 |  |  |
| Gallbladder and biliary tract cancer | Inflammatory bowel disease | Weighted median | 0.987 | 0.964 | 1.011 | 0.292 | 29 |  |  |
| Gallbladder and biliary tract cancer | Inflammatory bowel disease | Weighted mode | 0.989 | 0.968 | 1.010 | 0.307 | 29 |  |  |
| Gallbladder and biliary tract cancer | Inflammatory bowel disease | Maximum likelihood | 0.997 | 0.981 | 1.012 | 0.666 | 29 |  |  |
| Kidney cancer | Psoriasis | IVW | 0.998 | 0.965 | 1.032 | 0.890 | 14 | 0.975 | 0.369 |
| Kidney cancer | Psoriasis | MR Egger | 1.014 | 0.967 | 1.063 | 0.580 | 14 |  |  |
| Kidney cancer | Psoriasis | Weighted median | 1.003 | 0.958 | 1.049 | 0.910 | 14 |  |  |
| Kidney cancer | Psoriasis | Weighted mode | 1.004 | 0.962 | 1.047 | 0.865 | 14 |  |  |
| Kidney cancer | Psoriasis | Maximum likelihood | 0.998 | 0.965 | 1.032 | 0.890 | 14 |  |  |
| Leukaemia | Inflammatory bowel disease | IVW | 1.027 | 0.989 | 1.067 | 0.160 | 24 | 0.552 | 0.488 |
| Leukaemia | Inflammatory bowel disease | MR Egger | 0.997 | 0.911 | 1.092 | 0.952 | 24 |  |  |
| Leukaemia | Inflammatory bowel disease | Weighted median | 1.009 | 0.957 | 1.065 | 0.728 | 24 |  |  |
| Leukaemia | Inflammatory bowel disease | Weighted mode | 1.006 | 0.936 | 1.080 | 0.882 | 24 |  |  |
| Leukaemia | Inflammatory bowel disease | Maximum likelihood | 1.028 | 0.989 | 1.068 | 0.161 | 24 |  |  |
| Liver cancer | Asthma | IVW | 1.007 | 0.990 | 1.024 | 0.423 | 21 | 0.019 | 0.269 |
| Liver cancer | Asthma | MR Egger | 1.018 | 0.993 | 1.044 | 0.181 | 21 |  |  |
| Liver cancer | Asthma | Weighted median | 1.014 | 0.994 | 1.035 | 0.173 | 21 |  |  |
| Liver cancer | Asthma | Weighted mode | 1.015 | 0.996 | 1.035 | 0.143 | 21 |  |  |
| Liver cancer | Asthma | Maximum likelihood | 1.007 | 0.994 | 1.020 | 0.288 | 21 |  |  |
| Malignant skin melanoma | Inflammatory bowel disease | IVW | 1.000 | 0.976 | 1.024 | 0.976 | 35 | 0.455 | 0.188 |
| Malignant skin melanoma | Inflammatory bowel disease | MR Egger | 0.986 | 0.957 | 1.017 | 0.387 | 35 |  |  |
| Malignant skin melanoma | Inflammatory bowel disease | Weighted median | 0.994 | 0.962 | 1.027 | 0.708 | 35 |  |  |
| Malignant skin melanoma | Inflammatory bowel disease | Weighted mode | 0.993 | 0.966 | 1.021 | 0.630 | 35 |  |  |
| Malignant skin melanoma | Inflammatory bowel disease | Maximum likelihood | 1.000 | 0.976 | 1.024 | 0.976 | 35 |  |  |
| Malignant skin melanoma | Psoriasis | IVW | 0.985 | 0.958 | 1.013 | 0.294 | 35 | 0.074 | 0.174 |
| Malignant skin melanoma | Psoriasis | MR Egger | 1.001 | 0.966 | 1.038 | 0.952 | 35 |  |  |
| Malignant skin melanoma | Psoriasis | Weighted median | 0.992 | 0.960 | 1.024 | 0.608 | 35 |  |  |
| Malignant skin melanoma | Psoriasis | Weighted mode | 0.991 | 0.962 | 1.021 | 0.548 | 35 |  |  |
| Malignant skin melanoma | Psoriasis | Maximum likelihood | 0.985 | 0.960 | 1.010 | 0.227 | 35 |  |  |
| Mesothelioma | Atopic dermatitis | IVW | 0.994 | 0.983 | 1.005 | 0.314 | 31 | 0.156 | 0.330 |
| Mesothelioma | Atopic dermatitis | MR Egger | 0.987 | 0.968 | 1.006 | 0.174 | 31 |  |  |
| Mesothelioma | Atopic dermatitis | Weighted median | 0.989 | 0.974 | 1.005 | 0.180 | 31 |  |  |
| Mesothelioma | Atopic dermatitis | Weighted mode | 0.993 | 0.980 | 1.007 | 0.360 | 31 |  |  |
| Mesothelioma | Atopic dermatitis | Maximum likelihood | 0.994 | 0.984 | 1.004 | 0.255 | 31 |  |  |
| Multiple myeloma | Atopic dermatitis | IVW | 1.001 | 0.996 | 1.007 | 0.622 | 19 | 0.201 | 0.093 |
| Multiple myeloma | Atopic dermatitis | MR Egger | 0.996 | 0.988 | 1.004 | 0.352 | 19 |  |  |
| Multiple myeloma | Atopic dermatitis | Weighted median | 1.001 | 0.993 | 1.009 | 0.787 | 19 |  |  |
| Multiple myeloma | Atopic dermatitis | Weighted mode | 1.001 | 0.996 | 1.006 | 0.683 | 19 |  |  |
| Multiple myeloma | Atopic dermatitis | Maximum likelihood | 1.001 | 0.996 | 1.007 | 0.586 | 19 |  |  |
| Multiple myeloma | Inflammatory bowel disease | IVW | 0.999 | 0.992 | 1.007 | 0.891 | 19 | 0.546 | 0.677 |
| Multiple myeloma | Inflammatory bowel disease | MR Egger | 1.001 | 0.990 | 1.012 | 0.829 | 19 |  |  |
| Multiple myeloma | Inflammatory bowel disease | Weighted median | 1.001 | 0.992 | 1.011 | 0.831 | 19 |  |  |
| Multiple myeloma | Inflammatory bowel disease | Weighted mode | 1.001 | 0.992 | 1.009 | 0.858 | 19 |  |  |
| Multiple myeloma | Inflammatory bowel disease | Maximum likelihood | 0.999 | 0.992 | 1.007 | 0.891 | 19 |  |  |
| Non-Hodgkin lymphoma | Inflammatory bowel disease | IVW | 1.024 | 0.970 | 1.081 | 0.396 | 21 | 0.134 | 0.497 |
| Non-Hodgkin lymphoma | Inflammatory bowel disease | MR Egger | 1.067 | 0.938 | 1.215 | 0.337 | 21 |  |  |
| Non-Hodgkin lymphoma | Inflammatory bowel disease | Weighted median | 1.039 | 0.969 | 1.113 | 0.284 | 21 |  |  |
| Non-Hodgkin lymphoma | Inflammatory bowel disease | Weighted mode | 1.036 | 0.953 | 1.127 | 0.419 | 21 |  |  |
| Non-Hodgkin lymphoma | Inflammatory bowel disease | Maximum likelihood | 1.025 | 0.977 | 1.075 | 0.311 | 21 |  |  |
| Non-Hodgkin lymphoma | Psoriasis | IVW | 1.034 | 0.976 | 1.095 | 0.255 | 16 | 0.777 | 0.802 |
| Non-Hodgkin lymphoma | Psoriasis | MR Egger | 1.050 | 0.923 | 1.194 | 0.474 | 16 |  |  |
| Non-Hodgkin lymphoma | Psoriasis | Weighted median | 1.023 | 0.948 | 1.104 | 0.562 | 16 |  |  |
| Non-Hodgkin lymphoma | Psoriasis | Weighted mode | 1.019 | 0.911 | 1.139 | 0.750 | 16 |  |  |
| Non-Hodgkin lymphoma | Psoriasis | Maximum likelihood | 1.035 | 0.976 | 1.097 | 0.254 | 16 |  |  |
| Non-Hodgkin lymphoma | Rheumatoid arthritis | IVW | 1.032 | 0.975 | 1.092 | 0.272 | 13 | 0.847 | 0.664 |
| Non-Hodgkin lymphoma | Rheumatoid arthritis | MR Egger | 1.007 | 0.890 | 1.139 | 0.915 | 13 |  |  |
| Non-Hodgkin lymphoma | Rheumatoid arthritis | Weighted median | 1.024 | 0.948 | 1.105 | 0.552 | 13 |  |  |
| Non-Hodgkin lymphoma | Rheumatoid arthritis | Weighted mode | 1.027 | 0.924 | 1.142 | 0.631 | 13 |  |  |
| Non-Hodgkin lymphoma | Rheumatoid arthritis | Maximum likelihood | 1.033 | 0.975 | 1.094 | 0.269 | 13 |  |  |
| Non-melanoma skin cancer | Atopic dermatitis | IVW | 0.975 | 0.949 | 1.001 | 0.062 | 103 | 0.116 | 0.660 |
| Non-melanoma skin cancer | Atopic dermatitis | MR Egger | 0.963 | 0.909 | 1.021 | 0.211 | 103 |  |  |
| Non-melanoma skin cancer | Atopic dermatitis | Weighted median | 0.961 | 0.923 | 1.000 | 0.051 | 103 |  |  |
| Non-melanoma skin cancer | Atopic dermatitis | Weighted mode | 0.909 | 0.820 | 1.008 | 0.074 | 103 |  |  |
| Non-melanoma skin cancer | Atopic dermatitis | Maximum likelihood | 0.975 | 0.950 | 1.000 | 0.047 | 103 |  |  |
| Non-melanoma skin cancer | Psoriasis | IVW | 0.948 | 0.910 | 0.986 | 0.008 | 100 | 0.176 | 0.145 |
| Non-melanoma skin cancer | Psoriasis | MR Egger | 1.002 | 0.921 | 1.091 | 0.958 | 100 |  |  |
| Non-melanoma skin cancer | Psoriasis | Weighted median | 0.979 | 0.924 | 1.037 | 0.473 | 100 |  |  |
| Non-melanoma skin cancer | Psoriasis | Weighted mode | 1.006 | 0.911 | 1.111 | 0.906 | 100 |  |  |
| Non-melanoma skin cancer | Psoriasis | Maximum likelihood | 0.947 | 0.912 | 0.984 | 0.005 | 100 |  |  |
| Other malignant neoplasms | Atopic dermatitis | IVW | 0.972 | 0.942 | 1.002 | 0.063 | 85 | 0.198 | 0.523 |
| Other malignant neoplasms | Atopic dermatitis | MR Egger | 0.953 | 0.891 | 1.019 | 0.162 | 85 |  |  |
| Other malignant neoplasms | Atopic dermatitis | Weighted median | 0.964 | 0.919 | 1.012 | 0.137 | 85 |  |  |
| Other malignant neoplasms | Atopic dermatitis | Weighted mode | 0.905 | 0.803 | 1.020 | 0.106 | 85 |  |  |
| Other malignant neoplasms | Atopic dermatitis | Maximum likelihood | 0.972 | 0.944 | 1.000 | 0.053 | 85 |  |  |
| Other malignant neoplasms | Psoriasis | IVW | 0.945 | 0.904 | 0.987 | 0.011 | 81 | 0.425 | 0.365 |
| Other malignant neoplasms | Psoriasis | MR Egger | 0.983 | 0.893 | 1.082 | 0.725 | 81 |  |  |
| Other malignant neoplasms | Psoriasis | Weighted median | 0.976 | 0.915 | 1.042 | 0.474 | 81 |  |  |
| Other malignant neoplasms | Psoriasis | Weighted mode | 1.000 | 0.898 | 1.114 | 0.997 | 81 |  |  |
| Other malignant neoplasms | Psoriasis | Maximum likelihood | 0.945 | 0.904 | 0.987 | 0.011 | 81 |  |  |
| Other malignant neoplasms | Rheumatoid arthritis | IVW | 0.974 | 0.930 | 1.020 | 0.257 | 88 | 0.002 | 0.428 |
| Other malignant neoplasms | Rheumatoid arthritis | MR Egger | 1.009 | 0.914 | 1.114 | 0.860 | 88 |  |  |
| Other malignant neoplasms | Rheumatoid arthritis | Weighted median | 0.996 | 0.940 | 1.055 | 0.883 | 88 |  |  |
| Other malignant neoplasms | Rheumatoid arthritis | Weighted mode | 1.005 | 0.934 | 1.081 | 0.895 | 88 |  |  |
| Other malignant neoplasms | Rheumatoid arthritis | Maximum likelihood | 0.974 | 0.937 | 1.012 | 0.172 | 88 |  |  |
| Other pharynx cancer | Inflammatory bowel disease | IVW | 1.003 | 0.994 | 1.013 | 0.454 | 29 | 0.641 | 0.893 |
| Other pharynx cancer | Inflammatory bowel disease | MR Egger | 1.004 | 0.991 | 1.018 | 0.550 | 29 |  |  |
| Other pharynx cancer | Inflammatory bowel disease | Weighted median | 1.004 | 0.992 | 1.016 | 0.565 | 29 |  |  |
| Other pharynx cancer | Inflammatory bowel disease | Weighted mode | 1.004 | 0.993 | 1.015 | 0.489 | 29 |  |  |
| Other pharynx cancer | Inflammatory bowel disease | Maximum likelihood | 1.004 | 0.994 | 1.013 | 0.451 | 29 |  |  |
| Other pharynx cancer | Rheumatoid arthritis | IVW | 1.002 | 0.994 | 1.011 | 0.600 | 29 | 0.631 | 0.895 |
| Other pharynx cancer | Rheumatoid arthritis | MR Egger | 1.002 | 0.989 | 1.014 | 0.801 | 29 |  |  |
| Other pharynx cancer | Rheumatoid arthritis | Weighted median | 1.007 | 0.995 | 1.018 | 0.254 | 29 |  |  |
| Other pharynx cancer | Rheumatoid arthritis | Weighted mode | 1.004 | 0.995 | 1.013 | 0.421 | 29 |  |  |
| Other pharynx cancer | Rheumatoid arthritis | Maximum likelihood | 1.002 | 0.994 | 1.011 | 0.598 | 29 |  |  |
| Ovarian cancer | Atopic dermatitis | IVW | 0.925 | 0.663 | 1.292 | 0.649 | 15 | 0.321 | 0.692 |
| Ovarian cancer | Atopic dermatitis | MR Egger | 1.060 | 0.505 | 2.229 | 0.879 | 15 |  |  |
| Ovarian cancer | Atopic dermatitis | Weighted median | 0.926 | 0.593 | 1.445 | 0.735 | 15 |  |  |
| Ovarian cancer | Atopic dermatitis | Weighted mode | 1.128 | 0.597 | 2.133 | 0.716 | 15 |  |  |
| Ovarian cancer | Atopic dermatitis | Maximum likelihood | 0.924 | 0.670 | 1.273 | 0.627 | 15 |  |  |
| Ovarian cancer | Inflammatory bowel disease | IVW | 1.019 | 0.991 | 1.049 | 0.192 | 15 | 0.330 | 0.207 |
| Ovarian cancer | Inflammatory bowel disease | MR Egger | 1.056 | 0.995 | 1.121 | 0.095 | 15 |  |  |
| Ovarian cancer | Inflammatory bowel disease | Weighted median | 1.019 | 0.981 | 1.059 | 0.330 | 15 |  |  |
| Ovarian cancer | Inflammatory bowel disease | Weighted mode | 1.013 | 0.958 | 1.072 | 0.654 | 15 |  |  |
| Ovarian cancer | Inflammatory bowel disease | Maximum likelihood | 1.020 | 0.992 | 1.048 | 0.159 | 15 |  |  |
| Pancreatic cancer | Inflammatory bowel disease | IVW | 0.991 | 0.957 | 1.025 | 0.590 | 20 | 0.176 | 0.946 |
| Pancreatic cancer | Inflammatory bowel disease | MR Egger | 0.993 | 0.918 | 1.074 | 0.864 | 20 |  |  |
| Pancreatic cancer | Inflammatory bowel disease | Weighted median | 0.973 | 0.930 | 1.019 | 0.252 | 20 |  |  |
| Pancreatic cancer | Inflammatory bowel disease | Weighted mode | 0.973 | 0.922 | 1.027 | 0.331 | 20 |  |  |
| Pancreatic cancer | Inflammatory bowel disease | Maximum likelihood | 0.991 | 0.961 | 1.022 | 0.545 | 20 |  |  |
| Prostate cancer | Inflammatory bowel disease | IVW | 1.000 | 0.979 | 1.022 | 0.967 | 148 | 0.331 | 0.567 |
| Prostate cancer | Inflammatory bowel disease | MR Egger | 0.991 | 0.953 | 1.030 | 0.649 | 148 |  |  |
| Prostate cancer | Inflammatory bowel disease | Weighted median | 1.014 | 0.979 | 1.050 | 0.446 | 148 |  |  |
| Prostate cancer | Inflammatory bowel disease | Weighted mode | 1.005 | 0.970 | 1.041 | 0.788 | 148 |  |  |
| Prostate cancer | Inflammatory bowel disease | Maximum likelihood | 1.000 | 0.980 | 1.022 | 0.966 | 148 |  |  |
| Prostate cancer | Psoriasis | IVW | 0.997 | 0.945 | 1.051 | 0.904 | 148 | 0.198 | 0.683 |
| Prostate cancer | Psoriasis | MR Egger | 1.014 | 0.920 | 1.118 | 0.783 | 148 |  |  |
| Prostate cancer | Psoriasis | Weighted median | 0.952 | 0.875 | 1.035 | 0.245 | 148 |  |  |
| Prostate cancer | Psoriasis | Weighted mode | 0.955 | 0.870 | 1.050 | 0.343 | 148 |  |  |
| Prostate cancer | Psoriasis | Maximum likelihood | 0.997 | 0.947 | 1.049 | 0.900 | 148 |  |  |
| Tracheal, bronchus, and lung cancer | Inflammatory bowel disease | IVW | 1.058 | 1.000 | 1.119 | 0.048 | 22 | 0.958 | 0.754 |
| Tracheal, bronchus, and lung cancer | Inflammatory bowel disease | MR Egger | 1.091 | 0.898 | 1.325 | 0.393 | 22 |  |  |
| Tracheal, bronchus, and lung cancer | Inflammatory bowel disease | Weighted median | 1.068 | 0.989 | 1.153 | 0.094 | 22 |  |  |
| Tracheal, bronchus, and lung cancer | Inflammatory bowel disease | Weighted mode | 1.090 | 0.983 | 1.209 | 0.116 | 22 |  |  |
| Tracheal, bronchus, and lung cancer | Inflammatory bowel disease | Maximum likelihood | 1.059 | 1.001 | 1.121 | 0.046 | 22 |  |  |

MR: Mendelian randomization; OR: odds ratio; CI, confidence interval; IVW: inverse variance weight.

**Supplementary Table 5.** Causal effect of rheumatoid arthritis on Hodgkin lymphoma

| **Expose** | **Outcome** | **nSNP** | **MR methods** | **OR (95%CI)** | ***P*-Value** | **Heterogeneity** | **Horizontal pleiotropy** |
| --- | --- | --- | --- | --- | --- | --- | --- |
| Rheumatoid arthritis | Hodgkin lymphoma | 85 | IVW | 1.309(1.094,1.567) | 0.003 | 0.512 | 0.245 |
|  |  |  | MR-Egger | 1.570(1.103,2.236) | 0.014 |  |  |
|  |  |  | Weighted mode | 1.516(1.123,2.048) | 0.007 |  |  |
|  |  |  | Weighted median | 1.601(1.129,2.269) | 0.010 |  |  |
|  |  |  | Maximum likelihood | 1.313(1.095,1.575) | 0.003 |  |  |

MR: Mendelian randomization; OR: odds ratio; CI, confidence interval; IVW: inverse variance weight.
